# GWAS Identifies Novel Susceptibility Loci on 6p21.32 and 21q21.3 for Hepatocellular Carcinoma in Chronic Hepatitis B Virus Carriers

**DOI:** 10.1371/journal.pgen.1002791

**Published:** 2012-07-12

**Authors:** Shengping Li, Ji Qian, Yuan Yang, Wanting Zhao, Juncheng Dai, Jin-Xin Bei, Jia Nee Foo, Paul J. McLaren, Zhiqiang Li, Jingmin Yang, Feng Shen, Li Liu, Jiamei Yang, Shuhong Li, Shandong Pan, Yi Wang, Wenjin Li, Xiangjun Zhai, Boping Zhou, Lehua Shi, Xinchun Chen, Minjie Chu, Yiqun Yan, Jun Wang, Shuqun Cheng, Jiawei Shen, Weihua Jia, Jibin Liu, Jiahe Yang, Zujia Wen, Aijun Li, Ying Zhang, Guoliang Zhang, Xianrong Luo, Hongbo Qin, Minshan Chen, Hua Wang, Li Jin, Dongxin Lin, Hongbing Shen, Lin He, Paul I. W. de Bakker, Hongyang Wang, Yi-Xin Zeng, Mengchao Wu, Zhibin Hu, Yongyong Shi, Jianjun Liu, Weiping Zhou

**Affiliations:** 1 Department of Hepatobiliary Oncology, State Key Laboratory of Oncology in South China, Sun Yat-Sen University Cancer Center, Guangzhou, China; 2 State Key Laboratory of Genetic Engineering, Center for Fudan–VARI Genetic Epidemiology and MOE Key Laboratory of Contemporary Anthropology, School of Life Sciences, Fudan University, Shanghai, China; 3 Eastern Hepatobiliary Surgery Hospital, Second Military Medical University, Shanghai, China; 4 Human Genetics, Genome Institute of Singapore, A*STAR, Singapore, Singapore; 5 MOE Key Laboratory of Modern Toxicology, Jiangsu Key Laboratory of Cancer Biomarkers, Prevention, and Treatment, and State Key Laboratory of Reproductive Medicine, Nanjing Medical University, Nanjing, China; 6 Division of Genetics, Brigham and Women's Hospital, Harvard Medical School Boston and Broad Institute of Harvard and MIT, Cambridge, Massachusetts, United States of America; 7 Bio-X Institutes, Key Laboratory for the Genetics of Developmental and Neuropsychiatric Disorders (Ministry of Education), Shanghai Jiao Tong University, Shanghai, China; 8 Department of Hepatobiliary Surgery, Nantong Tumor Hospital, Nantong, China; 9 Department of Infection Diseases, Jiangsu Province Center for Disease Prevention and Control, Nanjing, China; 10 The Third People's Municipal Hospital of Shenzhen, Shenzhen, China; 11 No. 458 Hospital of Chinese People's Liberation Army, Guangzhou, China; 12 The Eighth Municipal People's Hospital of Guangzhou, Guangzhou, China; 13 State Key Laboratory of Molecular Oncology and Department of Etiology and Carcinogenesis, Cancer Institute and Hospital, Chinese Academy of Medical Sciences and Peking Union Medical College, Beijing, China; 14 Institutes for Nutritional Sciences, Shanghai Institute of Biological Sciences, Chinese Academy of Sciences, Shanghai, China; 15 Institutes of Biomedical Sciences, Fudan University, Shanghai, China; Imperial College London, United Kingdom

## Abstract

Genome-wide association studies (GWAS) have recently identified *KIF1B* as susceptibility locus for hepatitis B virus (HBV)–related hepatocellular carcinoma (HCC). To further identify novel susceptibility loci associated with HBV–related HCC and replicate the previously reported association, we performed a large three-stage GWAS in the Han Chinese population. 523,663 autosomal SNPs in 1,538 HBV–positive HCC patients and 1,465 chronic HBV carriers were genotyped for the discovery stage. Top candidate SNPs were genotyped in the initial validation samples of 2,112 HBV–positive HCC cases and 2,208 HBV carriers and then in the second validation samples of 1,021 cases and 1,491 HBV carriers. We discovered two novel associations at rs9272105 (*HLA-DQA1*/*DRB1*) on 6p21.32 (OR = 1.30, *P* = 1.13×10^−19^) and rs455804 (*GRIK1*) on 21q21.3 (OR = 0.84, *P* = 1.86×10^−8^), which were further replicated in the fourth independent sample of 1,298 cases and 1,026 controls (rs9272105: OR = 1.25, *P* = 1.71×10^−4^; rs455804: OR = 0.84, *P* = 6.92×10^−3^). We also revealed the associations of *HLA*-DRB1*0405 and 0901*0602, which could partially account for the association at rs9272105. The association at rs455804 implicates *GRIK1* as a novel susceptibility gene for HBV–related HCC, suggesting the involvement of glutamate signaling in the development of HBV–related HCC.

## Author Summary

Previous studies strongly suggest the importance of genetic susceptibility for hepatocellular carcinoma (HCC). However, the studies about genetic etiology on HBV–related HCC were limited. Our genome-wide association study included 523,663 autosomal SNPs in 1,538 HBV–positive HCC patients and 1,465 chronic HBV carriers for the discovery analysis. 2,112 HBV–positive HCC cases and 2,208 HBV carriers (the initial validation), and 1,021 cases and 1,491 HBV carriers (the second validation), were then analyzed for validation. The fourth independent samples of 1,298 cases and 1,026 controls were analyzed as replication. We discovered two novel associations at rs9272105 (*HLA-DQA1*/*DRB1*) on 6p21.32 and rs455804 (*GRIK1*) on 21q21.3. *HLA-DRB1* molecules play an important role in chronic HBV infection and progression to HCC. The association at rs455804 implicates *GRIK1* as a novel susceptibility gene for HBV–related HCC, suggesting the involvement of glutamate signaling in the development of HBV–related HCC.

## Introduction

Hepatocellular carcinoma (HCC) is the sixth common cancer and the third common cause of cancer mortality worldwide [1]. The incidence rate of HCC varies considerably in the world, with the highest in East, Southeast Asia and Sub-Saharan Africa, and China alone accounts for approximately half of HCC malignancies [1], [2]. Major risk factors for HCC are chronic infections with the hepatitis B or C viruses, and exposure to dietary aflatoxin B1. Hepatitis B virus (HBV) infection is particular important, because of its coherent distribution with the HCC prevalence [1], [2]. However, it is known that only a minority of chronic carriers of HBV develop HCC [3], and the chronic HBV carriers with a family history of HCC have a two-fold risk for HCC than those without the family history [4], strongly suggesting the importance of genetic susceptibility for HBV-related HCC.

A number of candidate genes were investigated by genetic association studies to evaluate their roles in the susceptibility to HCC [5]. However, the findings from these studies are inconclusive due to moderate evidence and lack of independent validation. Recently, a genome-wide association study (GWAS) of HBV-related HCC was performed [6], in which 355 HBV–positive HCC patients and 360 chronic HBV carriers were used for the genome-wide discovery analysis, and the top 45 SNPs from the discovery analysis were further evaluated in additional 1,962 HBV–positive HCC patients and 1,430 controls (both chronic HBV carriers and population controls) as well as 159 trios. The study identified *KIF1B* as a novel susceptibility locus (top SNP rs17401966) on 1p36.22. Further study with better design and bigger sample size was recommended for identifying additional susceptibility loci for HCC [7], [8]. These motivate us to carry out a GWAS with a large sample size in Chinese population to discover novel susceptibility loci for HCC.

## Results

We performed a genome-wide discovery analysis by analyzing 523,663 common autosomal SNPs in two independent cohorts of the Han Chinese: 480 cases and 484 controls from central China and 1058 cases and 981 controls from southern China (Table S1 and Figure S1). The principal component analysis (PCA) confirmed all the samples to be Chinese, but indicated moderate genetic mismatch between the cases and controls in the cohort of southern China (Figure S2). To minimize the effect of population stratification, we performed the genome-wide association analysis using PCA-based correction for population stratification. After the adjustment by the first principal component, the λgc of the genome-wide association results is 1.013 for the cohort of central China, 1.003 for the cohort of southern China and 1.012 for the combined samples. Furthermore, for all the three genome-wide analyses of central, southern and combined samples, the quantile-quantile (QQ) plot of the observed *P* values revealed a good overall fit with the null distribution (Figure S3). Taken together, these results clearly indicate that the final association results from our genome-wide discovery analysis are free of inflation effect due to population stratification.

The genome-wide discovery analysis revealed multiple suggestive associations (*P*<10^−5^) on 2q22.1, 6p21.32, 11p15.1 and 20q12 (Figure S4 and Table S2). To validate these findings, 39 SNPs were selected according to their overall association evidence in three GWAS analyses as well as their consistencies of association between the two independent GWAS samples (Central and Southern China) (see the Methods for the selection criteria). The 39 SNPs were genotyped in additional 2,112 HBV–positive HCC cases and 2,208 HBV carriers (Phase I validation) (Table S1). Of the 39 SNPs, only 3 (rs9272105 on 6p21.32, rs11148740 on 13q21.32 and rs455804 on 21q21.3) were validated, showing consistent association between the GWAS discovery and Phase I validation samples (Table S3). These 3 SNPs were then genotyped in additional 1,021 HBV–positive HCC cases and 1,491 HBV carriers (Phase II validation). The Phase II validation analysis (Table 1) confirmed the associations at rs9272105 on 6p21.32 (OR = 1.41, *P* = 7.63×10^−9^) and rs455804 on 21q21.3 (OR = 0.83, *P* = 3.63×10^−3^), but not the association at rs11148740 on 13q21.32 (Table S3).

**Table 1:** Summary of GWAS scan, validation, and replication studies for the 2 SNPs.

SNP	Study	Cases^b^ (n = 5,969)	Controls^b^ (n = 6,190)	MAF^c^		OR (95% CI) d	*P* d	*P*_Q^e^
				cases	controls			
6p21.32: rs9272105	GWAS Southern	348/456/251	239/450/286	0.55	0.48	1.28(1.13–1.46)	1.80E-04	
A/G a	GWAS Central	129/229/122	81/249/154	0.51	0.42	1.41(1.16–1.72)	6.45E-04	
	Joint GWAS	477/685/373	320/699/440	0.53	0.46	1.32(1.19–1.46)	2.26E-07	6.20E-01
	Validation 1	580/976/556	436/1118/653	0.51	0.45	1.24(1.14–1.35)	5.15E-07	
	Validation 2	278/420/242	260/770/459	0.52	0.43	1.41(1.25–1.58)	7.63E-09	
	Joint Validation	858/1396/798	696/1888/1112	0.51	0.44	1.30(1.21–.139)	8.73E-14	8.10E-02
	GWAS+Validation 1&2	1335/2081/1171	1016/2587/1552	0.52	0.45	1.30 (1.23–1.38)	1.13E-19	3.36E-01
	Replication	335/621/342	195/516/315	0.50	0.44	1.25(1.11–1.40)	1.71E-04	
	All combined	1670/2702/1513	1211/3103/1867	0.51	0.45	1.28(1.22–1.35)	5.24E-22	3.88E-01
21q21.3: rs455804	GWAS Southern	110/397/551	126/440/415	0.29	0.35	0.74(0.64–0.85)	3.37E-05	
A/C a	GWAS Central	47/215/218	56/215/213	0.32	0.34	0.94(0.76–1.16)	5.54E-01	
	Joint GWAS	157/612/769	182/655/628	0.30	0.35	0.81(0.72–0.90)	1.65E-04	1.09E-01
	Validation 1	201/888/1021	262/962/976	0.31	0.34	0.87(0.79–0.95)	1.62E-03	
	Validation 2	89/426/506	154/689/648	0.30	0.33	0.83(0.74–0.94)	3.63E-03	
	Joint Validation	290/1314/1527	416/1651/1624	0.30	0.34	0.85(0.79–0.92)	2.05E-05	5.41E-01
	GWAS+Validation 1&2	447/1926/2296	598/2306/2252	0.30	0.34	0.84 (0.79–0.89)	1.86E-08	3.15E-01
	Replication	123/530/645	106/476/444	0.30	0.34	0.84(0.74–0.95)	6.92E-03	
	All combined	570/2456/2941	704/2782/2696	0.30	0.34	0.84(0.80–0.89)	5.24E-10	3.14E-01

a Minor allele/major allele;

b Variant homozygote/Heterozygote/Wild type homozygote;

c Minor allele frequency (MAF);

d Adjusted by the first principal component;

e *P* value of heterogeneity based on Cochrane's *Q*test.

For both rs9272105 and rs455804, no heterogeneity of associations were observed among the GWAS and validation samples (*P*>0.05), and the associations in the combined GWAS and validation samples achieved genome-wide significance (*P*<5.0×10^−8^) (rs9272105: OR = 1.30, *P* = 1.13×10^−19^ and rs455804: OR = 0.84, *P* = 1.86×10^−8^) (Table 1). As a replication, these two SNPs were genotyped in the fourth independent samples of 1,298 cases and 1,026 controls from central China, which further confirmed the associations at rs9272105 (OR = 1.25, *P* = 1.71×10^−4^) and rs11148740 (OR = 0.84, *P* = 6.92×10^−3^) (Table 1). When combining all the five groups of samples, the two SNPs resulted in a 28% increased, and a 16% decreased risk for HCC development (rs9272105: OR = 1.28, *P* = 5.24×10^−22^ and rs455804: OR = 0.84, *P* = 5.24×10^−10^) (Table 1), respectively. The associations at the two SNPs remained genome-wide significant after adjusting for age, gender, smoking and drinking (Table S4A). Furthermore, stratification analysis by age, gender, smoking and drinking status revealed similar ORs for rs9272105 and rs455804 among subgroups, except that the association at rs9272105 showed a stronger effect in the non-smoking group than the smoking one (OR = 1.38 *vs.* 1.19, *P* for heterogeneity = 0.004) (Table S4B). Pair-wise interaction analysis among these two SNPs, smoking and drinking status did not reveal any significant interaction (data not shown). The samples used in the GWAS, validation and replication analyses are summarized in Table S1, and the multi-stage design of the whole study is shown in Figure S5.

We further investigated the association of HLA alleles in our GWAS samples through imputation. After QC filtering (see the Methods), 37 HLA alleles were successfully imputed, and 5 alleles showed nominal association (*P*<0.05) (Table S5 and Table 2). Further stepwise conditional analysis revealed that only two *DRB1* alleles showed independent associations (DRB1*0405: OR = 0.69, *P* = 6.18×10^−4^; DRB1*0901: OR = 0.82, *P* = 3.62×10^−3^) (Table 2). Conditioning on rs9272105 could abolish the associations of the *DRB1* alleles, and conditioning on the *DRB1* alleles could weaken, but not eliminate, the association at rs9272105 (Table 2). The haplotype analysis of rs9272105 and the two *DRB1* alleles revealed consistent result, showing that both the *DRB1* alleles sit on the haplotypes carrying the protective G allele of rs9272105 (Table S6). Taking together, there seems to be additional risk effect beyond the ones carried by the *DRB1* alleles.

**Table 2:** Summary of the association results of five imputed HLA alleles in the GWAS discovery samples.

CHR	HLA-allele	position	OR^a^	P^a^
6	HLA_DQA1_0301	32716284	0.85	4.39E-03
6	HLA_DQA1_0601	32716284	1.24	7.12E-03
6	HLA_DQB1_0401	32739039	0.71	4.49E-03
6	HLA_DRB1_0405	32660042	0.69	6.18E-04
6	HLA_DRB1_0901	32660042	0.85	1.46E-02
**Conditional on HLA_DRB1_0405**				
6	rs9272105	32707977	1.28	8.99E-06
6	HLA_DRB1_0901	32660042	0.82	3.62E-03
6	HLA_DQA1_0301	32716284	0.90	8.78E-02
6	HLA_DQA1_0601	32716284	1.22	1.35E-02
6	HLA_DQB1_0401	32739039	0.89	4.77E-01
**Conditional on HLA_DRB1_0405 and HLA_DRB1_0901**				
6	rs9272105	32707977	1.26	7.58E-04
6	HLA_DQA1_0301	32716284	1.14	2.35E-01
6	HLA_DQA1_0601	32716284	1.17	5.88E-02
6	HLA_DQB1_0401	32739039	0.87	4.13E-01
**Conditional on the five HLA alleles**				
6	rs9272105	32707977	1.25	2.08E-03
**Conditional on rs9272105**				
6	HLA_DRB1_0405	32660042	0.78	3.31E-02
6	HLA_DRB1_0901	32660042	1.02	7.90E-01
6	HLA_DQA1_0301	32716284	1.02	8.22E-01
6	HLA_DQA1_0601	32716284	1.08	4.04E-01
6	HLA_DQB1_0401	32739039	0.78	4.95E-02

a Adjusted by the first principal component.

We further explored whether the SNPs rs9272105 and rs455804 play any role in HBV infection. First, we compared the frequencies of these 2 SNPs between 408 non-symptomatic HBV carriers and 521 symptomatic chronic HBV patients from southern China (GWA scanned). The analysis revealed a protective effect at rs9272105 (OR = 0.80, *P* = 1.67×10^−2^) on the development of symptomatic chronic hepatitis B, but no association at rs455804 (Table S7A). Furthermore, we genotyped these 2 SNPs in 1,344 individuals with HBV nature clearance and compared their frequencies with those in 4,183 asymptomatic HBV carriers (all from the Central China). The analysis also revealed a protective association at rs9272105 for HBV chronic infection (OR = 0.88, *P* = 3.78×10^−3^) (Table S7B).

## Discussion

**Figure 1:**
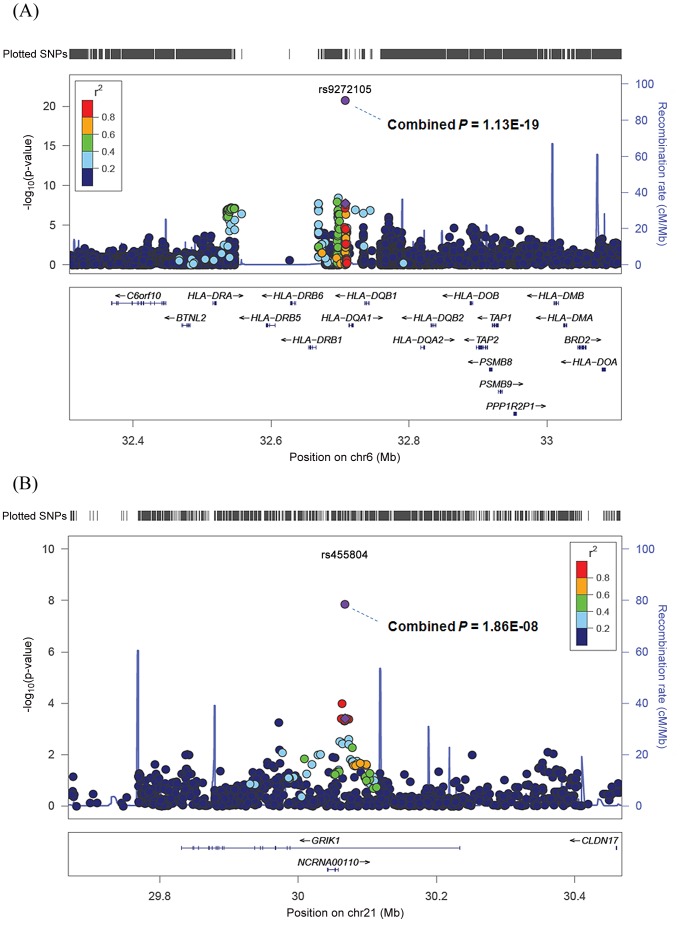
Regional plots of the susceptibility loci at rs9272105 on 6p and rs455804on 21q. rs9272105 on 6p (A); rs455804on 21q (B).The association result (−log_10_ *P*) is shown against the map position for each SNP within the region of 400 kb surrounding the validated association. The marker SNPs of the associations are shown in purple, and the *r* ^2^ values of the rest SNPs with the marker one are indicated in different colors based on the strengths. The annotated genes within the critical region of the association are shown in bottom.

SNP rs9272105 is located between *HLA-DQA1* and *HLA-DRB1* on 6p21.32 (Figure 1A). SNP imputation in the GWAS discovery samples revealed additional SNPs showing association, but rs9272105 remained to be the top SNP within the region (Figure 1A). The residual association at rs9272105 after conditioning the association effects of the *HLA* alleles DRB1*0405 and *0901 suggests that there may be additional risk effect beyond the DRB1 alleles in Chinese population. The associations of the *DRB1* alleles revealed by this study are consistent with the previous reports that *HLA-DQ/DR* alleles associated with HCC risk [9], [10]. In addition, we investigated the previously reported HBV infection-associated SNPs rs3077, rs9277535, rs7453920, and rs2856718 within the HLA *DP/DQ* region [11], [12] with HCC development in our GWAS samples. By imputation, we found the evidence of the association at rs9277535 with HCC (rs9277535: OR = 0.85, *P* = 7.9×10^−3^). However, there is no linkage disequilibrium (LD) between rs9277535 and our SNP rs9272105 (r^2^ = 0.016 according the HapMap CHB+JPT samples), suggesting that the associations at rs9277535 and rs9272105 may be independent.

The *HLA-DQ* locus has also been shown to be associated with HCV-related HCC in a Japanese GWAS (rs9275572, OR = 1.30, *P* = 9.38×10^−9^) [13]. SNPs rs9275572 and rs9272105 are 79 kb away from each other and in weak LD (*D*′ = 0.43, *r* ^2^ = 0.08 in the HapMap CHB samples). The SNP rs9275572 did not show any association with HBV-related HCC in our GWAS discovery samples (OR = 0.93, *P* = 0.24) (Table S8 and Figure S6B). In addition to *HLA-DQ*, *MICA* (rs2596542) on 6p21.33 and *DEPDC5* (rs1012068) on 22q12.3 were also identified as independent susceptibility loci for HCV-related HCC in Japanese population [13], [14]. But, our GWAS discovery analysis did not reveal any supportive evidence for these two loci (rs2596542: OR = 1.06, *P* = 0.36; and rs1012068: OR = 1.06, *P* = 0.37) (Table S8 and Figure S6C and S6D). We also evaluated the power of our GWAS discovery samples and found that our samples should have sufficient power for detecting the previously reported associations at rs9275572 (power = 94%), rs2596542 (power = 92%) and rs1012068 (power = 94%). Taken together, the disparity of associations may suggest the different genetic background of the susceptibilities for HCV- and HBV-related HCC. Further studies will be required to confirm the genetic heterogeneity of HCV- and HBV-related HCC.

The association of rs9272105 (*HLA-DQA1*/*DRB1*) with HBV infection is consistent with the extensive reports on the association of *HLA-DRB1* with HBV infection where both protective and risk *DRB1* alleles for HBV infection and outcome were identified [11], [12], [15]–[19]. Intriguingly, our study has revealed that the variant allele of rs9272105 showed a protective effect for HBV infection (OR = 0.88) and the progression to chronic symptomatic hepatitis B, but a risk effect for the development of HCC (OR = 1.30). Further studies will be needed to demonstrate whether the opposite associations of HBV infection and HBV-related HCC progression at rs9272105 are due to different causal variants within the HLA class II region.

SNP rs455804 is located within the first intron of *GRIK1* that is the only gene within the LD region of the association (Figure 1B), strongly implicating *GRIK1* as a novel susceptibility gene for HBV-related HCC. SNP imputation of the region did not reveal any SNPs that showed stronger association than rs455804. *GRIK1* encodes CLUR5, which is involved in the glutamate signaling, as one of the ionotropic glutamate receptor, kainite 1 protein (GLUR5), a subunit of ligand-activated channels and involved in glutamate signaling. Our discovery of the association of *GRIK1* with HCC has enhanced the emerging evidences for the important role of glutamate signaling pathway in cancer development. Glutamate has been shown to play a central role in the malignant phenotype of gliomas through multiple molecular mechanisms [20]. Inhibition of glutamate release and/or glutamate receptor activity can inhibit the proliferation and/or invasion of tumor cells in breast cancer [21], laryngeal cancer [22], and pancreatic cancer [23], and ionotrpic glutamate receptor (GLUR6) was also suggested to play a tumor-suppressor role in gastric cancer [24]. Recently, the exome sequencing analysis revealed that *GRIN2A* (encoding the ionotrpic glutamate receptor (N-methyl D-aspartate) subunit 2A) was mutated in 33% of melanoma tumors, clearly indicating the involvement of glutamate signaling in melanoma development. Finally, SNPs within *GRIK1* have also been found significantly associated with paclitaxel response in NCI60 cancer cell lines, and may play a role in the cellular response to paclitaxel treatment in cancer [25]. Consistent with the previous observations, our discovery of *GRIK1* as a HBV-related HCC susceptibility gene has suggested the importance of glutamate signaling in HBV-related HCC development, and, although still speculative, has highlighted the glutamate signaling pathway as a potentially novel target for the treatment of HCC.

We also assessed the previously reported susceptibility locus *KIF1B* on 1p36.22 (rs17401966) for HBV-related HCC [6]. Our GWAS discovery analysis did reveal the consistent result for the association at rs17401966, but the strength of association in our GWAS discovery sample (OR = 0.90) is much weaker than the previously reported one (OR = 0.61) (Table S8). SNP imputation in our GWAS discovery samples did not reveal any stronger association than the association at rs17401966 within the LD region surrounding the 1p36.22 locus (Figure S6A).

Previous studies have clearly shown the existence of subpopulation structure of Chinese Han population along the north-south axis, and further demonstrated that geographic matching can be used as a good surrogate for genetic matching, and PCA-based correction is very effective in controlling the inflation effect of population stratification [26]. In the current study, all the cases and controls were matched by their geographic origin of residence. Moreover, the GWAS discovery samples were from central and southern China, while all the validation and replication samples were from central China. Our PCA analysis indicates that while there was mild population stratification in the sample of southern China, the cases and controls from central China were well matched without any indication of population stratification. In our study, the PCA-based correction was used in the GWAS analysis, and all the validation and replication analyses were from central China. Therefore, our findings should be free of adverse effect of population stratification in Chinese population.

In conclusion, the current GWAS identified two biologically plausible, novel loci on 6p21.32 and 21q21 for HBV-related HCC. These findings highlight the importance of HLA-DQ/DR molecules and glutamate signaling in the development of HBV-related HCC.

## Methods

### Patient samples

The genome-wide discovery analysis was performed by genotyping 731,442 SNPs in 1,575 HBV positive HCC patients and 1,490 HBV positive controls derived from two independent case-control cohorts of 500 cases and 500 controls from Central China (Shanghai) and 1,075 cases and 990 controls from Southern China (Guangdong). The first stage validation samples included 2,112 HBV–positive cases and 2,208 HBV–positive controls recruited from Jiangsu. The second stage validation samples consisted of 1,021 HBV–positive cases and 1,491HBV carriers recruited from Shanghai. The replication samples of 1,298 HBV–positive cases and 1,026 HBV carriers were recruited from Central China (Shanghai and Jiangsu). (Table S1 and Figure 1) All the samples are Han Chinese and partially participated in the previously published studies [27], [28]. The diagnosis of HCC was confirmed by a pathological examination and/or α-fetoprotein elevation (>400 ng/ml) combined with imaging examination (Magnetic resonance imaging, MRI and/or computerized tomography, CT). Because HCV infection is rare in Chinese, we excluded HCC with HCV infection. Cancer-free HBV+ control subjects from central China were recruited from those receiving routine physical examinations in local hospitals or those participating in the community-based screening for the HBV/HCV markers and frequency-matched for age, gender, and geographic regions to each set of the HCC patients. Almost all these community-based controls are asymptomatic HBV carriers. Similarly, cancer-free control subjects from southern China are all HBV+, and 408 of them were asymptomatic HBV carriers and 521 were symptomatic chronic hepatitis B patients. All the HBV+ controls were positive for both HBsAg and antibody to hepatitis B core antigen (anti-HBc), and negative for anti-HCV.

We also recruited a HBV natural clearance cohort form Jiangsu Province (Zhangjiagang and Changzhou cities) through a population based screening for the HBV/HCV markers in 2004 and 2009, respectively (58,142 persons). Subjects with HBV natural clearance were negative for HBsAg and anti-HCV, positive for both antibody to hepatitis B surface antigen (anti-HBs) and anti-HBc. About 9,610 subjects with HBV natural clearance were identified. No history of hepatitis B vaccination was reported for these people. Then, we randomly selected 1,344 HBV natural clearance people without self-reported history of cancer in the current study. The age for the 1,344 people were 52.6±10.2 years, and 217(16.2%) were females.

We collected smoking and drinking information through interviews. Those who had smoked an average of less than 1 cigarette per day and less than 1 year in their lifetime were defined as nonsmokers; otherwise, they were considered as smokers. Individuals were classified as alcohol drinkers if they drank at least twice a week and continuously for one year during their lifetime; otherwise, they were defined as nondrinkers. At recruitment, the informed consent was obtained from each subject, and this study was approved by the Institutional Review Boards of each participating institution.

### Quality control in GWA Scan

We performed standard quality control on the raw genotyping data to filter both unqualified samples and SNPs. The samples with overall genotype completion rates <95% were excluded from further analysis (26 subjects). Eight subjects were excluded as they showed discrepancy between the recorded and genetically inferred genders. An additional 21 duplicates or probable familial relatives were excluded based on the IBD analysis implemented in PLINK (all PI_HAT>0.25). SNPs were excluded when they fit the following criteria: (i) not mapped on autosomal chromosomes; (ii) had a call rate <95% in all GWA samples or in either of Central cohort study or Southern study samples; (iii) had minor allele frequency (MAF) <0.05 in either of Central cohort study or Southern study samples; and (iv) genotype distributions deviated from those expected by Hardy-Weinberg equilibrium (*P*<1×10^−5^ in either of Central cohort study or Southern study samples). We detected population outliers and stratification using a principal component analysis (PCA) based method. After removing MHC SNPs on chromosome 6 from 25–37 Mb, PCA was performed by using common autosomal SNPs with low LD (*r* ^2^<0.2) in the reference samples of the HapMap project (YRI (n = 90), CEU (n = 90), CHB (n = 45) and JPT (n = 44)) as the internal controls and our 3,010 participants of the GWAS discovery samples (after removal of samples with low call rates, ambiguous gender, and familial relationships). Projection onto the two multidimensional scaling axes is shown in Figure S2A. 7 outliers (more than 6 standard deviations) were identified and excluded. Finally, 523,663 autosomal SNPs in 1,538 cases and 1,465 controls, consisting of 480 cases and 484 controls from Central China and 1,058 cases and 981 controls from Southern China, were retained for association testing (Table S1).

### SNPs selection and genotyping in validation phases

SNPs for the first stage validation were selected based on the following criteria: (i) SNP had *P* joint≤1.0×10^−4^ in the analysis of the combined GWA samples or either the Central China sample or the Southern China sample, and had a consistent association in the two participant studies, meaning that the ORs from the two samples are both either above or below 1; (ii) only SNP with the lowest *P* value was selected when multiple SNPs showed a strong LD (*r* ^2^≥0.8). As a result, a total of 39 SNPs were included in the first stage validation. 3 SNPs that were significantly associated with HCC risk in the first validation stage were further genotyped in the second stage validation samples. Genotyping in the two validation samples were done by using the iPLEX platform (Sequenom) or the TaqMan assays (Applied Biosystems). The primers and probes were available upon request (Table S9). Laboratory technicians who performed genotyping experiments were blinded to case/control status. For TaqMan assay, ten percent of random samples were repeated, and the reproducibility was 100%. The 2 validated SNPs were genotyped in another independent replication using the same method.

### Statistical analysis

Population structure was evaluated by the PCA in the software package EIGENSTRAT 3.0 [26]. PCA revealed one significant (*P*<0.05) eigenvector which was included in the logistic regression with other covariates of age, gender, smoking and drinking status for both the genome-wide discovery analysis and the joint analysis of the combined discovery and replication samples. Ancestral origin checking by PCA confirmed all the samples to be Han Chinese and further demonstrated moderate genetic stratification between the cases and the controls of the Southern cohort (Figure S2). The genome-wide association analysis was therefore performed in logistic regression using PCA-based correction for population stratification and by treating the samples of two cohorts as independent studies. The genomic-control inflation factor (λgc) after adjustment by the first PC was calculated for the Central cohort samples (λgc = 1.013), the Southern cohort samples (λgc = 1.003) and the combined GWAS discovery samples (λgc = 1.012). Consistently, the QQ plot of the observed *P* values also showed a minimal inflation of genome-wide association results due to population stratification (Figure S3).

Statistical analyses were performed by using PLINK 1.07 [29] and R 2.11.1. The Manhattan plot of −log_10_ *P* was generated using Haploview (v4.1) [30]. Untyped genotypes were imputed in the GWAS discovery samples by using IMPUTE2 [31] and the haplotype information from the 1000 Genomes Project (ASN samples as the reference set) and HapMap3 (CHB and JPT samples as the reference samples). The regional plot of association was created by using an online tool, LocusZoom 1.1. *P* value was two-sided, and OR presented in the manuscript was estimated by using additive model and logistic regression analyses if not specified.

To impute classical HLA alleles, we used 180 phased haplotypes from the HapMap CHB and JPT samples as our reference panel. This panel comprised dense SNP data and HLA allele types at 4-digit resolution for the HLA class I (HLA-A, B, C) and II (DQA1, DQB1 and DRB1) genes as previously described [32]. Genotypes, probability and allelic dosages were then imputed separately in the two discovery samples of Central and Southern Chinese using the BEAGLE program. Association testing was performed by using a logistic regression model on the best-guessed genotypes and allelic dosages. The results were checked for consistency between the two methods, and the results from best-guessed genotypes were presented.

## Supporting Information

Figure S1 The map of China. The regions of the sample collection were highlighted in red.
(DOCX)

Figure S2 Plots of principal components from the PCA for genetic matching. (A) a-b: plot of the first two PCs from the PCA of GWAS (central and southern) samples and the HapMap individuals. (B) plots between the 1^st^∼8^th^ PCs, which derived from PCA of 964 Central samples. (C) plots between the 1^st^∼8^th^ PCs, which derived from PCA of 2039 Southern samples. (D) plots between the 1^st^∼8^th^ PCs, which derived from PCA of 3033 Central and Southern samples.
(DOCX)

Figure S3 Quantile–Quantile plot. (A) QQ plot of Central Samples. (B) QQ plot of Southern Samples. (C) QQ plot of combined GWAS Samples.
(DOCX)

Figure S4 Manhattan plot of the genome-wide P values of association. Association was assessed using logistic regression analysis with adjustment for the first principal components of population stratification. (A) Manhattan plot for Central Samples. (B) Manhattan plot for Southern Samples. (C) Manhattan plot for combined GWAS Samples.
(DOCX)

Figure S5 Workflow of the study.
(DOCX)

Figure S6 Regional plots of 4 interested regions.
(DOCX)

Table S1 Summary description of the samples used in this study.
(DOCX)

Table S2 Summary of all the SNPs with P values less than 10^−4^.
(DOCX)

Table S3 A: Associations of the 39 fast-track replicated SNPs from the GWAS scan. B: Associations of 39 SNPs in GWAS scan and validations.
(DOCX)

Table S4 Adjusted and stratified analyses of the 2 validated SNPs.
(DOCX)

Table S5 Results of all the HLA alleles that have been successfully imputed after quality controls.
(DOCX)

Table S6 The haplotype analysis of the two DRB1 alleles and rs9272105.
(DOCX)

Table S7 A: Association of two SNPs in the subjects with asymptomatic and symptomatic HBV infection from the southern Chinese cohort. B: Association of rs9272105 and rs455804 in asymptomatic HBV carriers and HBV natural clearance samples from central China.
(DOCX)

Table S8 Association results of rs17401966, rs2596542, rs1012068, and rs9275572 in the GWAS samples.
(DOCX)

Table S9 Information of primers and probes for the 39 fast-track replicated SNPs of the GWAS scan.
(DOCX)
