# Supplementary material for: GWAS Identifies Novel Susceptibility Loci on 6p21.32 and 21q21.3 for Hepatocellular Carcinoma in Chronic Hepatitis B Virus Carriers
Source: PLoS Genet. 2012 Jul 12;8(7):e1002791. doi: 10.1371/journal.pgen.1002791 (PMC3395595; doi:10.1371/journal.pgen.1002791)
Supplement: Figure S2 — Plots of principal components from the PCA for genetic matching. (A) a-b: plot of the first two PCs from the PCA of GWAS (central and southern) samples and the HapMap individuals. (B) plots between the 1st∼8th PCs, which derived from PCA of 964 Central samples. (C) plots between the 1st∼8th PCs, which derived from PCA of 2039 Southern samples. (D) plots between the 1st∼8th PCs, which derived from PCA of 3033 Central and Southern samples. (DOCX) [file pgen.1002791.s002.docx]

**Figure S2** **Plots of principal components from the PCA for genetic matching**

1. a-b: plot of the first two PCs from the PCA of GWAS (central and southern) samples and the HapMap individuals.
2. plots between the 1^st^~ 8^th^ PCs, which derived from PCA of 964 Central samples.
3. plots between the 1^st^~ 8^th^ PCs, which derived from PCA of 2039 Southern samples.
4. plots between the 1^st^~ 8^th^ PCs, which derived from PCA of 3033 Central and Southern samples
5. **GWAS (central and southern) samples and the HapMap samples**

**
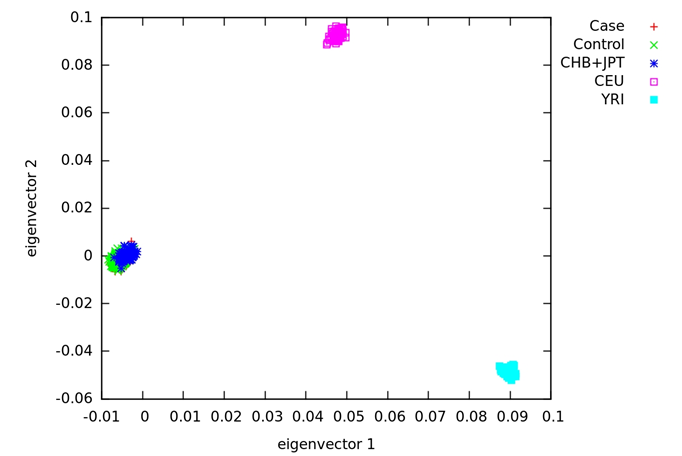

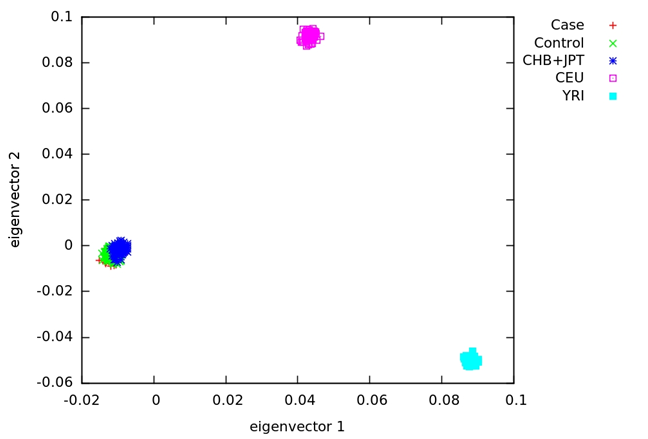
a.** pc1 vs.pc2 (Central samples and HapMap samples) **b.**pc1 vs.pc2 (Southern samples and HapMap samples)

1. **Central Samples**

**
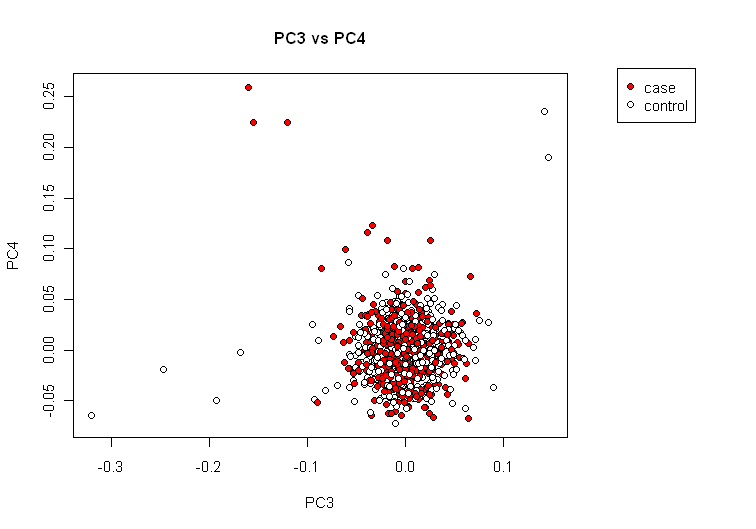

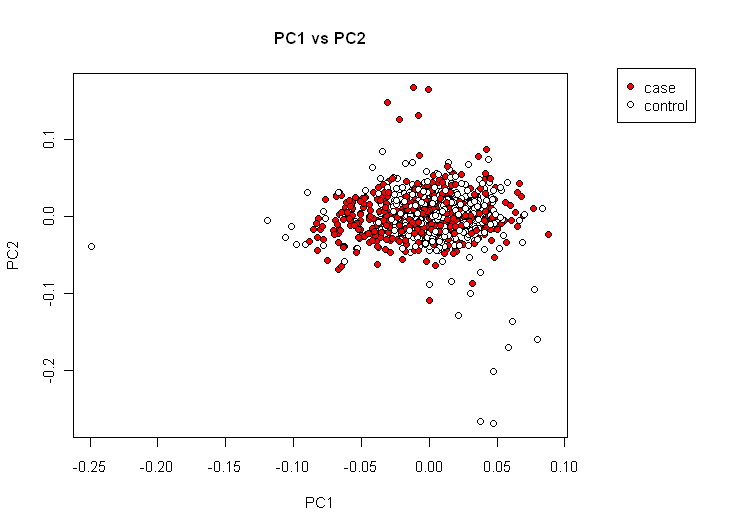
**

**
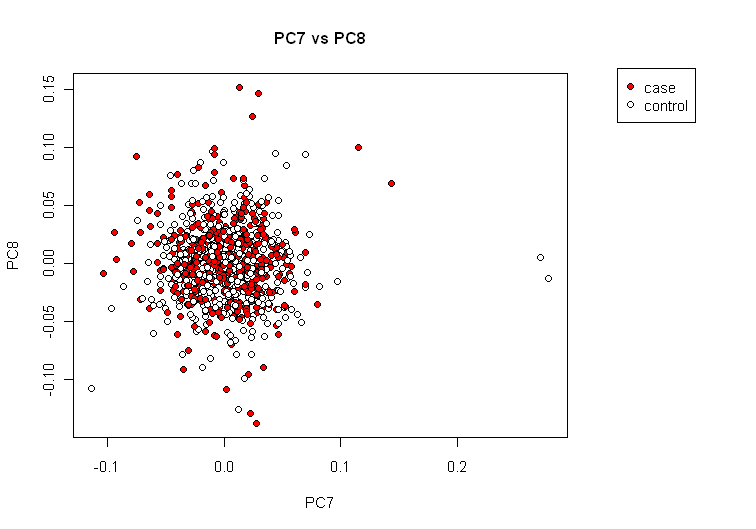

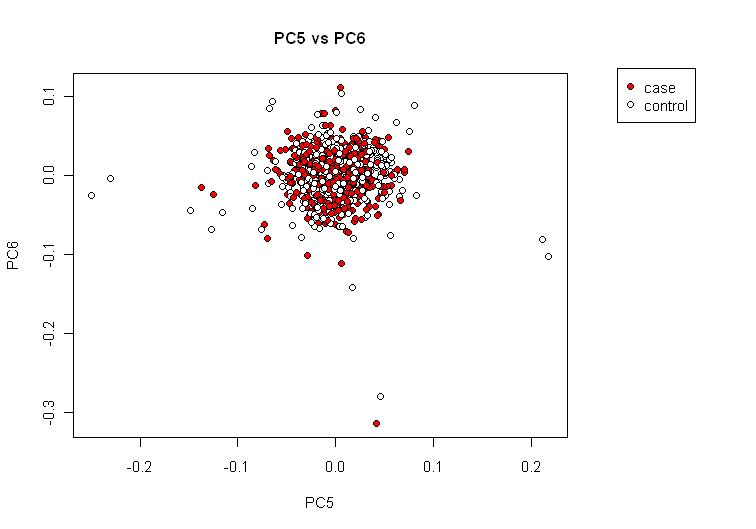
**

1. **Southern Samples**

**
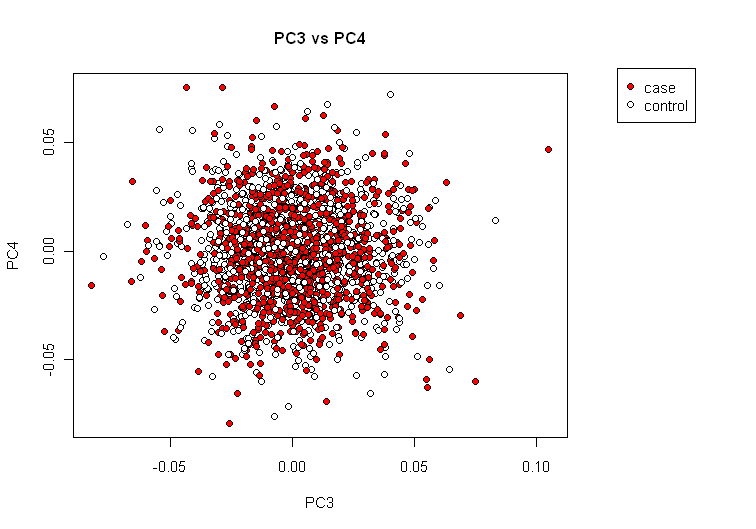

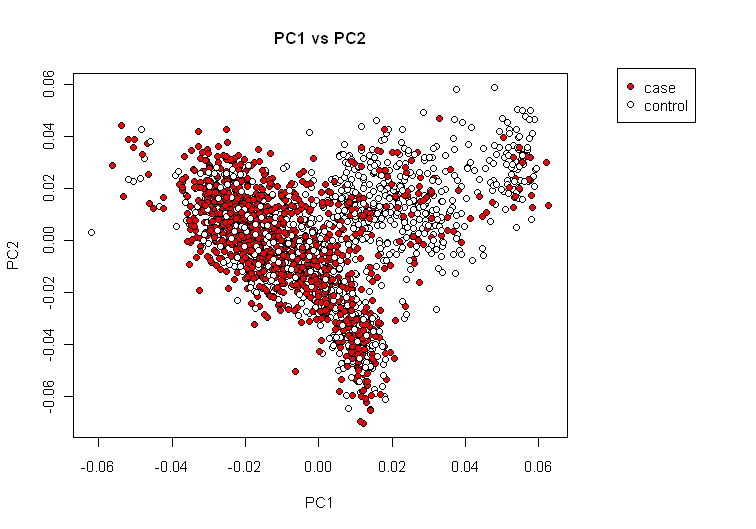
**

**
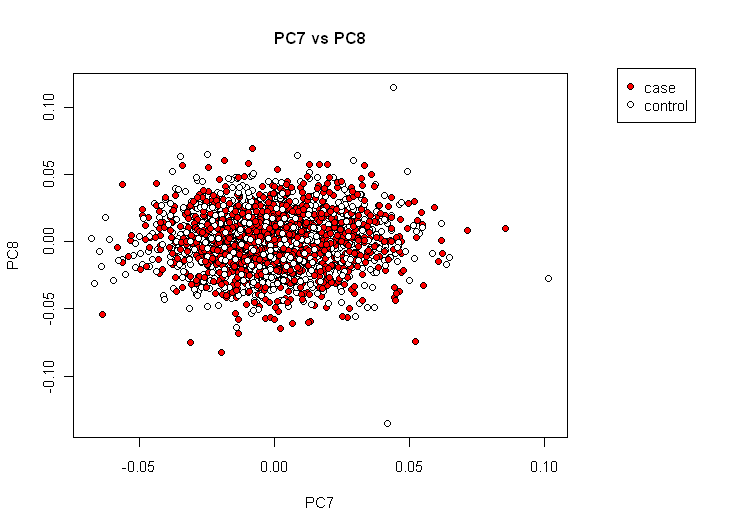

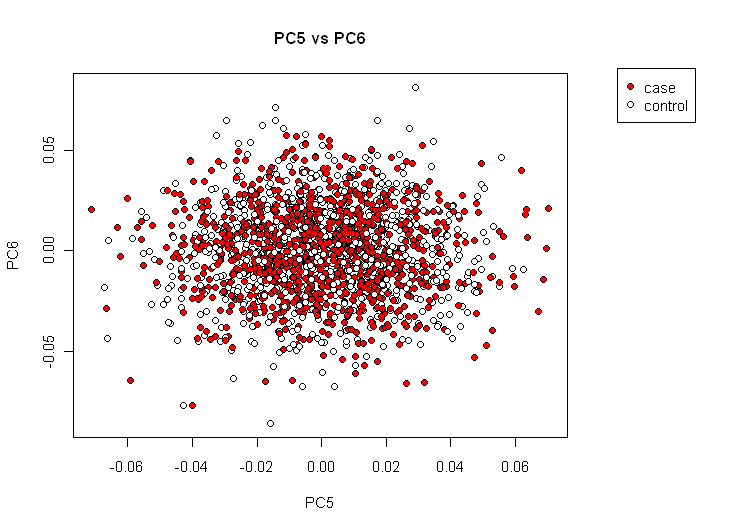
**

1. **Combined Central and Southern samples**

**
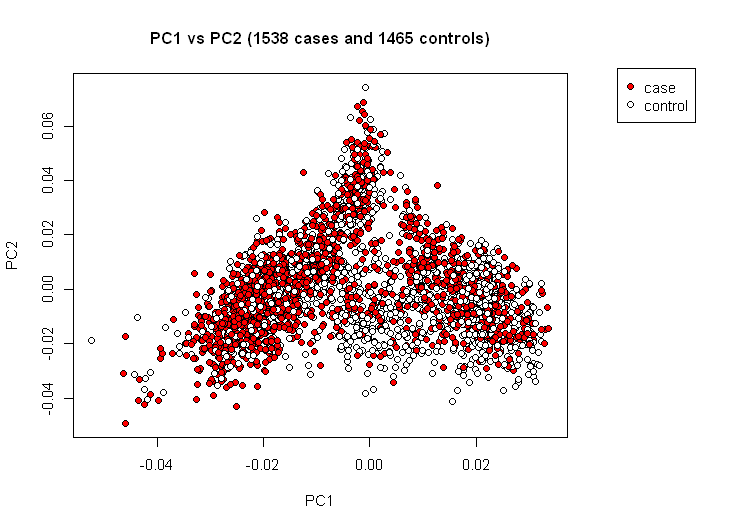

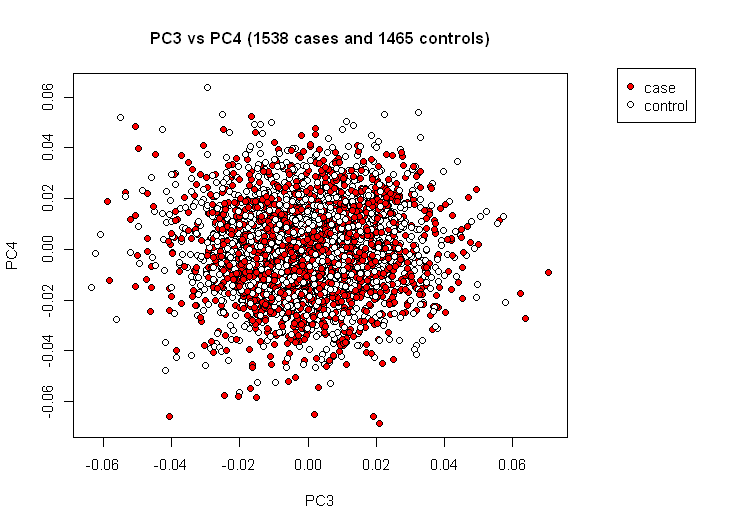
**

**
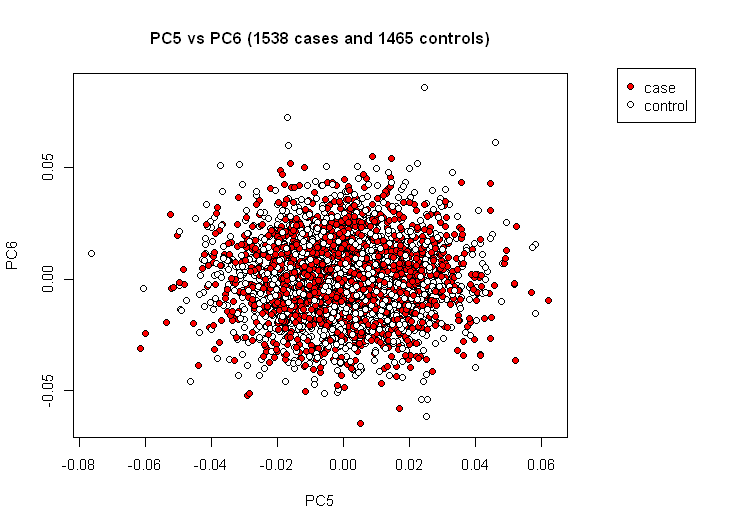

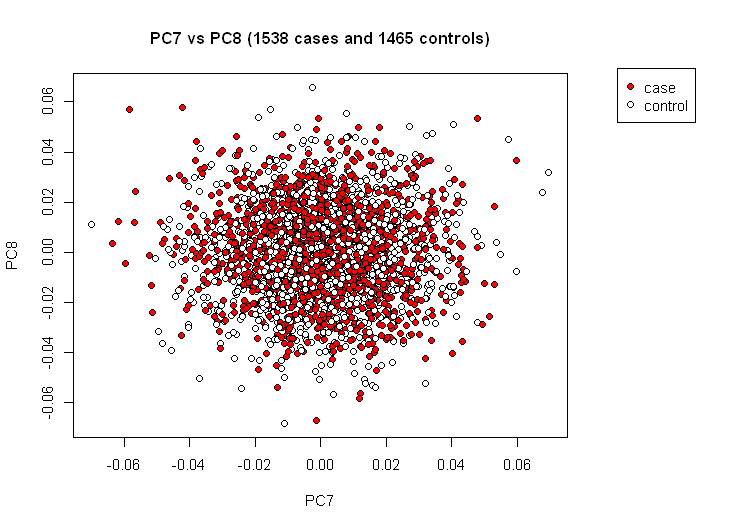
**
