# Supplementary material for: GWAS Identifies Novel Susceptibility Loci on 6p21.32 and 21q21.3 for Hepatocellular Carcinoma in Chronic Hepatitis B Virus Carriers
Source: PLoS Genet. 2012 Jul 12;8(7):e1002791. doi: 10.1371/journal.pgen.1002791 (PMC3395595; doi:10.1371/journal.pgen.1002791)
Supplement: Figure S3 — Quantile–Quantile plot. (A) QQ plot of Central Samples. (B) QQ plot of Southern Samples. (C) QQ plot of combined GWAS Samples. (DOCX) [file pgen.1002791.s003.docx]

**Figure S3 Quantile-Quantile plot**

1. QQ plot of Central samples.


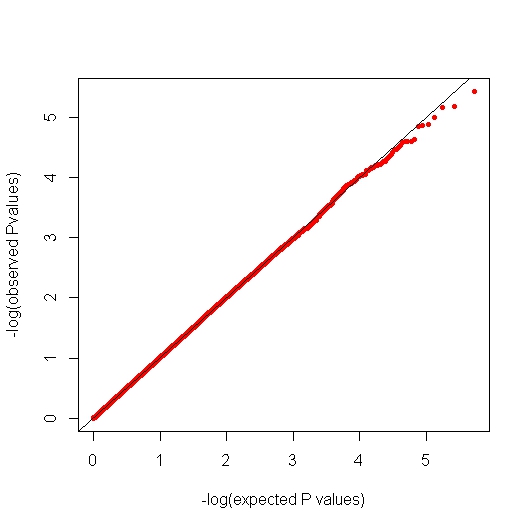


λ=1.013

1. QQ plot of Southern Samples


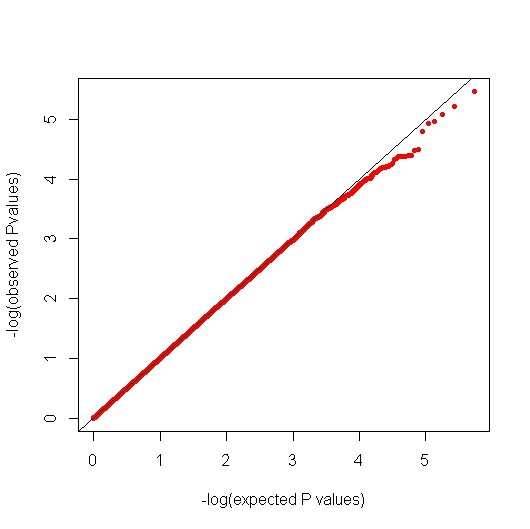


λ=1.003

1. QQ plot of combined GWAS Samples

**
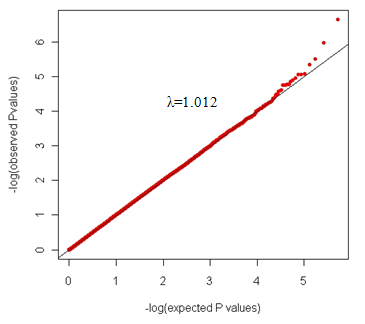
**
