# Supplementary material for: GWAS Identifies Novel Susceptibility Loci on 6p21.32 and 21q21.3 for Hepatocellular Carcinoma in Chronic Hepatitis B Virus Carriers
Source: PLoS Genet. 2012 Jul 12;8(7):e1002791. doi: 10.1371/journal.pgen.1002791 (PMC3395595; doi:10.1371/journal.pgen.1002791)

**Figure S4** Manhattan plot of the genome-wide *P* values of association. Association was assessed using logistic regression analysis with adjustment for the first principal components of population stratification.

1. Manhattan plot for Central Samples.


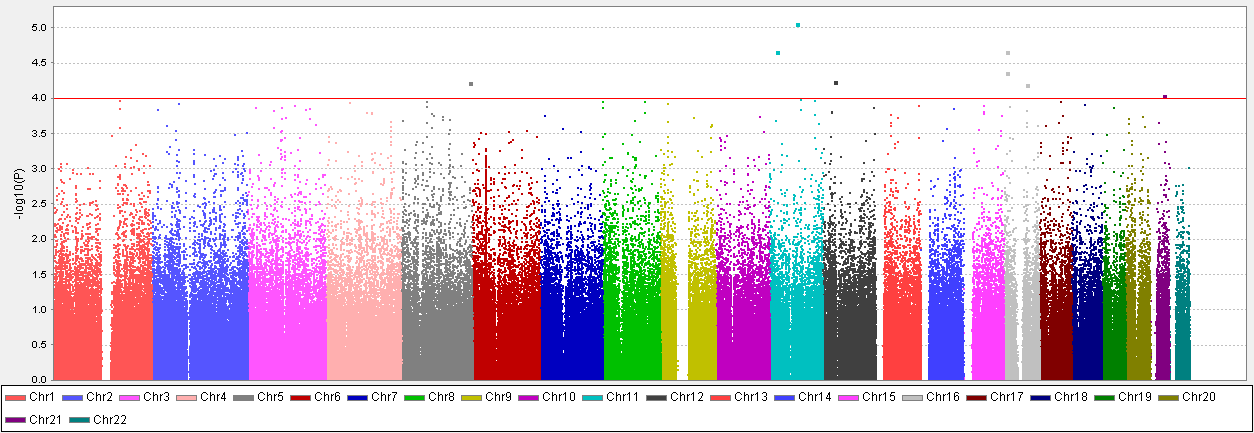


1. Manhattan plot for Southern Samples


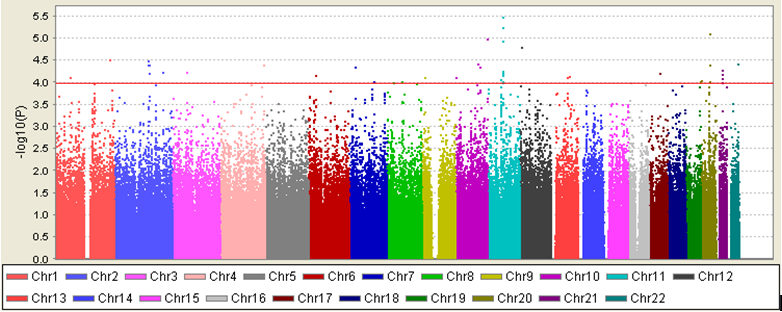


**C.** Manhattan plot for combined GWAS Samples
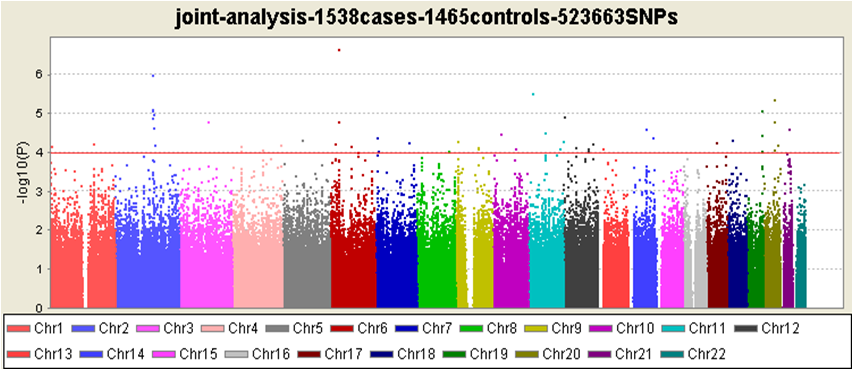

Supplement: Figure S4 — Manhattan plot of the genome-wide P values of association. Association was assessed using logistic regression analysis with adjustment for the first principal components of population stratification. (A) Manhattan plot for Central Samples. (B) Manhattan plot for Southern Samples. (C) Manhattan plot for combined GWAS Samples. (DOCX) [file pgen.1002791.s004.docx]
