# Supplementary figures and images for: GWAS Identifies Novel Susceptibility Loci on 6p21.32 and 21q21.3 for Hepatocellular Carcinoma in Chronic Hepatitis B Virus Carriers
Source: PLoS Genet. 2012 Jul 12;8(7):e1002791. doi: 10.1371/journal.pgen.1002791 (PMC3395595; doi:10.1371/journal.pgen.1002791)

**Figure S5** Workflow of the study


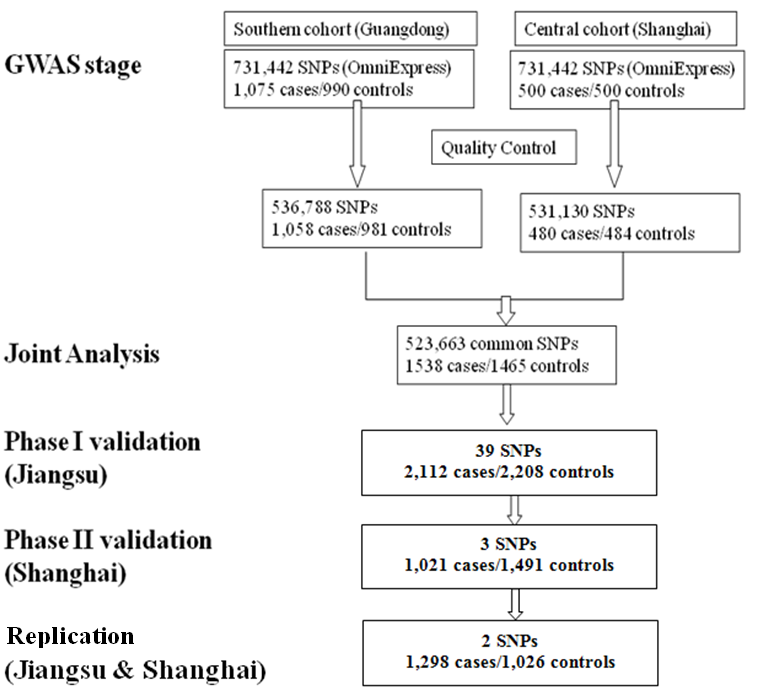

Supplement: Figure S5 — Workflow of the study. (DOCX) [file pgen.1002791.s005.docx]
