# Supplementary material for: GWAS Identifies Novel Susceptibility Loci on 6p21.32 and 21q21.3 for Hepatocellular Carcinoma in Chronic Hepatitis B Virus Carriers
Source: PLoS Genet. 2012 Jul 12;8(7):e1002791. doi: 10.1371/journal.pgen.1002791 (PMC3395595; doi:10.1371/journal.pgen.1002791)
Supplement: Figure S6 — Regional plots of 4 interested regions. (DOCX) [file pgen.1002791.s006.docx]

**Figure S6** Regional plots of 4 interested regions. Results (-log_10_ *P*) are shown for SNPs for the region of ﬂanking 400 kb on either side of the marker SNPs. The marker SNPs are shown in purple and the *r* ^2^ values of the rest of the SNPs are indicated by different colors. The genes within the interested region are annotated and shown as arrows.

(A)

-log_10_(p-value)

(B)

-log_10_(p-value)

(C)

-log_10_(p-value)

(D)

-log_10_(p-value)


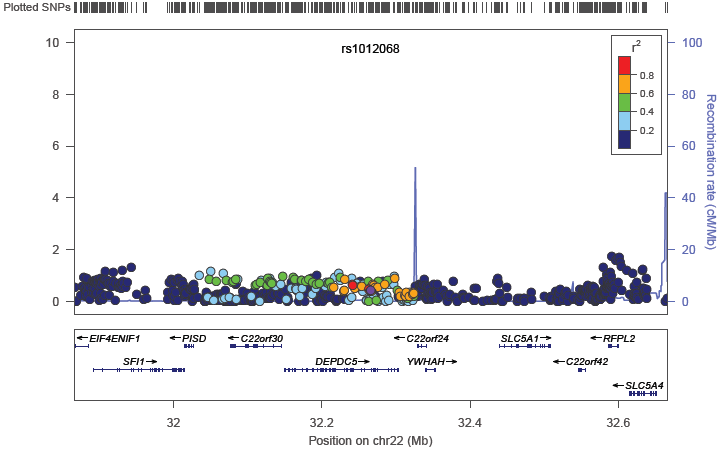


-log_10_(p-value)
