# Supplementary material for: GWAS Identifies Novel Susceptibility Loci on 6p21.32 and 21q21.3 for Hepatocellular Carcinoma in Chronic Hepatitis B Virus Carriers
Source: PLoS Genet. 2012 Jul 12;8(7):e1002791. doi: 10.1371/journal.pgen.1002791 (PMC3395595; doi:10.1371/journal.pgen.1002791)
Supplement: Table S1 — Summary description of the samples used in this study. (DOCX) [file pgen.1002791.s007.docx]

**Table S1** Summary description of the samples used in this study

| **Variables** | **GWAS scan** | | | **Validation 1** | | | **Validation 2** | | | **Replication** | | |
| --- | --- | --- | --- | --- | --- | --- | --- | --- | --- | --- | --- | --- |
|  | **Case (n=1,538)** | **Control (n=1,465)** |  | **Case**  **(n=2,112)** | **Control (n=2,208)** |  | **Case (n=1,021)** | **Control**  **(n=1,491)** |  | **Case**  **(n=1,298)** | **Control**  **(n=1,026)** |  |
| **Age (Mean ± S.D.)** | 49.26±11.37 | 48.82±11.66 |  | 51.83±10.42 | 51.78±10.89 |  | 50.83±10.90 | 51.97±12.31 |  | 53.13±11.17 | 52.43±11.24 |  |
| **Gender (%)** |  |  |  |  |  |  |  |  |  |  |  |  |
| **Male** | 1363(88.62) | 1135(77.47) |  | 1824(86.36) | 1903(86.19) |  | 835(81.78) | 1151(77.20) |  | 1077(82.97) | 820(79.92) |  |
| **Female** | 175(11.38) | 330(22.53) |  | 288(13.64) | 305(13.81) |  | 186(18.22) | 340(22.80) |  | 221(17.03) | 206(20.08) |  |
| **Smoking Status (%)** |  |  |  |  |  |  |  |  |  |  |  |  |
| **Smokers** | 624(40.57) | 802(54.74) |  | 1030(48.77) | 1238(56.07) |  | 440(43.10) | 756(50.70) |  | 643(49.54) | 514(50.10) |  |
| **Non-smokers** | 914(59.43) | 663(45.26) |  | 1082(51.23) | 970(43.93) |  | 581(56.90) | 735(49.30) |  | 655(50.46) | 512(49.90) |  |
| **Drinking Status (%)** |  |  |  |  |  |  |  |  |  |  |  |  |
| **Drinkers** | 529(34.40) | 599(40.89) |  | 931(44.08) | 929(42.07) |  | 306(29.97) | 475(31.86) |  | 497(38.29) | 370(36.06) |  |
| **Non-drinkers** | 1009(65.60) | 866(59.11) |  | 1181(55.92) | 1279(57.93) |  | 715(70.03) | 1016(68.14) |  | 801(61.71) | 656(63.94) |  |
