# Supplementary material for: GWAS Identifies Novel Susceptibility Loci on 6p21.32 and 21q21.3 for Hepatocellular Carcinoma in Chronic Hepatitis B Virus Carriers
Source: PLoS Genet. 2012 Jul 12;8(7):e1002791. doi: 10.1371/journal.pgen.1002791 (PMC3395595; doi:10.1371/journal.pgen.1002791)
Supplement: Table S2 — Summary of all the SNPs with P values less than 10−4. (DOCX) [file pgen.1002791.s008.docx]

**Table S2** Summary of all the SNPs with *P* values less than 10^-4^

|  | **Southern** |  |  |  | **Central** |  |  |  | **Joint** |  |
| --- | --- | --- | --- | --- | --- | --- | --- | --- | --- | --- |
| **SNP ID** | **Chr. position** | ***P* value** |  | **SNP ID** | **Chr. position** | ***P* value** |  | **SNP ID** | **Chr. position** | ***P* value** |
| rs7119426 | Chr11:66406188 | 3.45E-06 |  | rs3905886 | Chr3:96254895 | 3.66E-06 |  | rs9272105 | Chr6:32707977 | 2.26E-07 |
| rs10160758 | Chr11:66429655 | 5.99E-06 |  | rs10896464 | Chr11:68835488 | 6.45E-06 |  | rs1714259 | Chr2:141648942 | 1.06E-06 |
| rs3092194 | Chr20:39549621 | 8.20E-06 |  | rs1073547 | Chr4:164629006 | 6.78E-06 |  | rs3825023 | Chr11:18227245 | 3.14E-06 |
| rs9418952 | Chr10:134903594 | 1.08E-05 |  | rs368007 | Chr20:7033280 | 9.86E-06 |  | rs3092194 | Chr20:39549621 | 4.41E-06 |
| rs3741364 | Chr11:66207754 | 1.19E-05 |  | rs1539834 | Chr18:32037780 | 1.27E-05 |  | rs1249458 | Chr2:141645695 | 8.34E-06 |
| rs11053534 | Chr12:7420967 | 1.61E-05 |  | rs4771628 | Chr13:108726509 | 1.34E-05 |  | rs402071 | Chr19:59459691 | 8.64E-06 |
| rs6693489 | Chr1:227369459 | 3.21E-05 |  | rs7927923 | Chr11:77657062 | 1.38E-05 |  | rs7424161 | Chr2:141658705 | 8.76E-06 |
| rs1714259 | Chr2:141648942 | 3.30E-05 |  | rs11237451 | Chr11:77703107 | 2.25E-05 |  | rs1317530 | Chr2:146377211 | 1.10E-05 |
| rs4411227 | Chr10:94821503 | 3.96E-05 |  | rs3825023 | Chr11:18227245 | 2.44E-05 |  | rs11053534 | Chr12:7420967 | 1.24E-05 |
| rs4391 | Chr22:47032443 | 3.96E-05 |  | rs10812030 | Chr9:24136203 | 2.49E-05 |  | rs2222233 | Chr2:141636413 | 1.35E-05 |
| rs1317530 | Chr2:146377211 | 4.20E-05 |  | rs752499 | Chr4:124747408 | 2.49E-05 |  | rs9272219 | Chr6:32710247 | 1.66E-05 |
| rs6029710 | Chr20:39563500 | 4.20E-05 |  | rs481168 | Chr11:123099450 | 2.52E-05 |  | rs10933971 | Chr3:110106078 | 1.71E-05 |
| rs13142457 | Chr4:184941379 | 4.21E-05 |  | rs3105672 | Chr16:7995739 | 2.90E-05 |  | rs9273012 | Chr6:32719619 | 1.73E-05 |
| rs7424161 | Chr2:141658705 | 4.25E-05 |  | rs10793294 | Chr11:77674051 | 3.14E-05 |  | rs6029710 | Chr20:39563500 | 1.73E-05 |
| rs2189494 | Chr7:24173553 | 4.53E-05 |  | rs1669715 | Chr8:583641 | 3.41E-05 |  | rs7744001 | Chr6:32734064 | 1.73E-05 |
| rs7093712 | Chr10:103071277 | 4.62E-05 |  | rs1669716 | Chr8:583707 | 3.41E-05 |  | rs1515238 | Chr2:146395873 | 2.45E-05 |
| rs396331 | Chr21:30065381 | 5.48E-05 |  | rs2726595 | Chr8:59872804 | 3.80E-05 |  | rs743417 | Chr21:34269830 | 2.51E-05 |
| rs10750790 | Chr11:66338786 | 5.60E-05 |  | rs1806533 | Chr4:124729124 | 3.99E-05 |  | rs7149261 | Chr14:69945266 | 2.66E-05 |
| rs10931978 | Chr2:202483300 | 6.00E-05 |  | rs8102 | Chr16:55976226 | 4.47E-05 |  | rs10160758 | Chr11:66429655 | 3.21E-05 |
| rs10791894 | Chr11:66436314 | 6.12E-05 |  | rs4151117 | Chr16:55974635 | 4.70E-05 |  | rs7119426 | Chr11:66406188 | 3.26E-05 |
| rs6803449 | Chr3:59985809 | 6.12E-05 |  | rs356220 | Chr4:90860363 | 4.84E-05 |  | rs2666261 | Chr10:33396145 | 3.44E-05 |
| rs6504626 | Chr17:45374890 | 6.34E-05 |  | rs6536728 | Chr4:164552830 | 5.29E-05 |  | rs17305346 | Chr19:59487148 | 3.83E-05 |
| rs1515238 | Chr2:146395873 | 6.39E-05 |  | rs2287904 | Chr5:64617808 | 5.39E-05 |  | rs234601 | Chr14:96146269 | 4.31E-05 |
| rs4930390 | Chr11:66316599 | 6.47E-05 |  | rs1570295 | Chr14:64380120 | 5.63E-05 |  | rs2189638 | Chr7:11589417 | 4.32E-05 |
| rs6591226 | Chr11:66432566 | 6.59E-05 |  | rs2964057 | Chr5:173787543 | 5.65E-05 |  | rs7226750 | Chr18:20390828 | 4.93E-05 |
| rs455804 | Chr21:30068040 | 6.64E-05 |  | rs4662631 | Chr2:129501193 | 5.90E-05 |  | rs16877319 | Chr5:79225932 | 5.04E-05 |
| rs10791889 | Chr11:66250401 | 6.99E-05 |  | rs7977334 | Chr12:29801645 | 5.90E-05 |  | rs1822387 | Chr9:11607265 | 5.46E-05 |
| rs9272105 | Chr6:32707977 | 7.28E-05 |  | rs7197658 | Chr16:8000442 | 5.92E-05 |  | rs12364540 | Chr11:133365249 | 5.46E-05 |
| rs7337666 | Chr13:77971249 | 7.42E-05 |  | rs12325667 | Chr16:8005213 | 6.09E-05 |  | rs7215223 | Chr17:38856821 | 5.75E-05 |
| rs7541201 | Chr1:63250946 | 7.87E-05 |  | rs10057851 | Chr5:64601017 | 6.18E-05 |  | rs1465387 | Chr7:130881572 | 5.77E-05 |
| rs11148740 | Chr13:66743336 | 7.88E-05 |  | rs7217181 | Chr17:45347475 | 6.20E-05 |  | rs7313883 | Chr12:112525230 | 6.03E-05 |
| rs9423430 | Chr10:5512332 | 7.88E-05 |  | rs356168 | Chr4:90893454 | 6.34E-05 |  | rs7512378 | Chr1:166091758 | 6.21E-05 |
| rs7022407 | Chr9:14015101 | 8.01E-05 |  | rs1703927 | Chr8:603620 | 6.63E-05 |  | rs7739131 | Chr6:21835127 | 6.32E-05 |
| rs459617 | Chr21:30062624 | 8.56E-05 |  | rs12514074 | Chr5:64584390 | 6.64E-05 |  | rs13142457 | Chr4:184941379 | 6.47E-05 |
| rs2870434 | Chr11:55828133 | 8.67E-05 |  | rs3812045 | Chr5:38632878 | 6.81E-05 |  | rs6721330 | Chr2:149567152 | 6.51E-05 |
| rs6134424 | Chr20:1198463 | 9.46E-05 |  | rs243054 | Chr2:60443449 | 6.86E-05 |  | rs7262054 | Chr20:56054718 | 6.84E-05 |
| rs4142393 | Chr20:39511499 | 9.67E-05 |  | rs17727363 | Chr3:64708480 | 7.00E-05 |  | rs9341799 | Chr6:80621238 | 7.18E-05 |
| rs3087751 | Chr20:1095410 | 9.71E-05 |  | rs2736990 | Chr4:90897564 | 7.34E-05 |  | rs907314 | Chr4:37930848 | 7.20E-05 |
| rs17313716 | Chr7:103388618 | 9.80E-05 |  | rs11868833 | Chr17:74621639 | 7.43E-05 |  | rs7518471 | Chr1:4728466 | 7.27E-05 |
| rs481296 | Chr11:66176130 | 9.85E-05 |  | rs2298620 | Chr9:24136436 | 7.45E-05 |  | rs11168830 | Chr12:47710801 | 7.86E-05 |
| rs4430089 | Chr8:63535187 | 9.87E-05 |  | rs4382457 | Chr8:124662671 | 7.48E-05 |  | rs1927084 | Chr9:88765298 | 7.97E-05 |
|  |  |  |  | rs8180209 | Chr4:90863477 | 7.58E-05 |  | rs9792548 | Chr9:88762903 | 8.03E-05 |
|  |  |  |  | rs10967875 | Chr9:27299659 | 8.59E-05 |  | rs7036455 | Chr9:88743917 | 8.27E-05 |
|  |  |  |  | rs12642830 | Chr4:164602649 | 8.67E-05 |  | rs481168 | Chr11:123099450 | 8.30E-05 |
|  |  |  |  | rs12021649 | Chr1:165401957 | 8.77E-05 |  | rs3129595 | Chr13:21458282 | 8.31E-05 |
|  |  |  |  | rs1460238 | Chr8:105892565 | 8.81E-05 |  | rs12782065 | Chr10:89350918 | 8.46E-05 |
|  |  |  |  | rs9790095 | Chr3:96323544 | 8.90E-05 |  | rs6538797 | Chr12:96198599 | 8.51E-05 |
|  |  |  |  | rs748800 | Chr1:165403352 | 8.90E-05 |  | rs13135130 | Chr4:118732713 | 9.13E-05 |
|  |  |  |  | rs3740677 | Chr11:77605684 | 9.02E-05 |  | rs4142393 | Chr20:39511499 | 9.22E-05 |
|  |  |  |  | rs11747988 | Chr5:64561277 | 9.14E-05 |  | rs4833022 | Chr4:37927687 | 9.37E-05 |
|  |  |  |  | rs984038 | Chr3:65737292 | 9.16E-05 |  | rs7967235 | Chr12:96198249 | 9.44E-05 |
|  |  |  |  | rs8019503 | Chr14:19526162 | 9.25E-05 |  | rs7254645 | Chr19:59467201 | 9.47E-05 |
|  |  |  |  | rs9308060 | Chr4:164611675 | 9.28E-05 |  | rs10505427 | Chr8:123887125 | 9.78E-05 |
|  |  |  |  | rs17290842 | Chr4:60234254 | 9.34E-05 |  | rs10269275 | Chr7:13640704 | 9.86E-05 |
|  |  |  |  | rs2277188 | Chr9:72354457 | 9.58E-05 |  |  |  |  |
|  |  |  |  | rs743417 | Chr21:34269830 | 9.63E-05 |  |  |  |  |
|  |  |  |  | rs932869 | Chr14:81304805 | 9.76E-05 |  |  |  |  |
|  |  |  |  | rs10756902 | Chr9:17700876 | 9.94E-05 |  |  |  |  |
