# Supplementary material for: GWAS Identifies Novel Susceptibility Loci on 6p21.32 and 21q21.3 for Hepatocellular Carcinoma in Chronic Hepatitis B Virus Carriers
Source: PLoS Genet. 2012 Jul 12;8(7):e1002791. doi: 10.1371/journal.pgen.1002791 (PMC3395595; doi:10.1371/journal.pgen.1002791)
Supplement: Table S3 — A: Associations of the 39 fast-track replicated SNPs from the GWAS scan. B: Associations of 39 SNPs in GWAS scan and validations. (DOCX) [file pgen.1002791.s009.docx]

**Table S3**

**A:** Associations of the 39 fast-track replicated SNPs from the GWAS scan

| **Chr.**  **(Cytoband)** | **SNP** | **Associated Gene** | **Allele ^a^** | **MAF ^b^** | | **OR_c ^c^** | ***P*_c ^c^** | **OR_s ^c^** | ***P*_s ^c^** | **OR_all ^d^** | ***P*_all ^d^** |
| --- | --- | --- | --- | --- | --- | --- | --- | --- | --- | --- | --- |
|  |  |  |  | **Case** | **Control** |  |  |  |  |  |  |
| 1p36.32 | rs7518471 | LOC100287848 | C>T | 0.08 | 0.06 | 1.50 | 1.37E-02 | 1.58 | 1.82E-03 | 1.54 | 7.27E-05 |
| 1q24.2 | rs7512378 | ADCY10 | A>G | 0.25 | 0.20 | 1.24 | 4.90E-02 | 1.33 | 4.28E-04 | 1.3 | 6.21E-05 |
| 2q22.1 | rs7424161 | LRP1B | C>T | 0.23 | 0.28 | 0.81 | 5.23E-02 | 0.73 | 4.25E-05 | 0.76 | 8.76E-06 |
| 2q22.3 | rs1317530 | LOC100131409 | C>T | 0.43 | 0.38 | 1.19 | 6.31E-02 | 1.34 | 4.20E-05 | 1.28 | 1.10E-05 |
| 2q23.1 | rs6721330 | KIF5C | C>A | 0.24 | 0.20 | 1.46 | 1.02E-03 | 1.23 | 1.09E-02 | 1.31 | 6.51E-05 |
| 3q13.13 | rs10933971 | TRAT1 | C>T | 0.47 | 0.41 | 1.40 | 5.28E-04 | 1.21 | 4.92E-03 | 1.27 | 1.71E-05 |
| 4p14 | rs907314 | TBC1D1 | C>T | 0.14 | 0.18 | 0.66 | 1.81E-03 | 0.78 | 7.86E-03 | 0.74 | 7.20E-05 |
| 5q14.1 | rs16877319 | CMYA5 | T>C | 0.08 | 0.06 | 1.59 | 1.03E-02 | 1.53 | 1.68E-03 | 1.55 | 5.04E-05 |
| 5q35.2 | rs2964057 | HMP19/LOC100127922 | A>G | 0.24 | 0.20 | 1.60 | 5.65E-05 | 1.12 | 1.70E-01 | 1.26 | 5.59E-04 |
| 6p21.32 | rs9272105 | HLA-DRB1 | G>A | 0.53 | 0.46 | 1.37 | 7.78E-04 | 0.77 | 7.28E-05 | 1.32 | 2.26E-07 |
| 6p22.3 | rs7739131 | FLJ22536 | C>T | 0.39 | 0.34 | 1.23 | 3.19E-02 | 1.27 | 7.14E-04 | 1.26 | 6.32E-05 |
| 6q14.1 | rs9341799 | SH3BGRL2/ELOVL4 | C>T | 0.38 | 0.43 | 0.74 | 1.59E-03 | 0.84 | 9.18E-03 | 0.8 | 7.18E-05 |
| 7p21.3 | rs2189638 | THSD7A | A>G | 0.41 | 0.35 | 1.36 | 1.21E-03 | 1.21 | 6.94E-03 | 1.26 | 4.32E-05 |
| 7q32.3 | rs1465387 | PODXL | A>G | 0.28 | 0.23 | 1.30 | 1.20E-02 | 1.28 | 1.66E-03 | 1.28 | 5.77E-05 |
| 9p23 | rs1822387 | PTPRD | C>T | 0.44 | 0.40 | 1.35 | 1.99E-03 | 1.21 | 6.00E-03 | 1.25 | 5.46E-05 |
| 9q21.33 | rs7036455 | ZCCHC6 | T>C | 0.44 | 0.48 | 0.79 | 1.37E-02 | 0.81 | 2.13E-03 | 0.8 | 8.27E-05 |
| 10p11.22 | rs2666261 | ITGB1 | C>T | 0.24 | 0.20 | 1.39 | 3.72E-03 | 1.28 | 2.62E-03 | 1.32 | 3.44E-05 |
| 10q23.2 | rs12782065 | MINPP1/PAPSS2 | C>T | 0.10 | 0.13 | 0.82 | 1.65E-01 | 0.67 | 1.18E-04 | 0.71 | 8.46E-05 |
| 11p15.1 | rs3825023 | SAA2 | C>T | 0.15 | 0.10 | 1.90 | 2.44E-05 | 1.32 | 6.29E-03 | 1.48 | 3.14E-06 |
| 11q13.1 | rs10160758 | PC | T>C | 0.24 | 0.20 | 1.10 | 3.62E-01 | 1.46 | 5.99E-06 | 1.32 | 3.21E-05 |
| 11q13.3 | rs10896464 | MYEOV/CCND1 | C>T | 0.24 | 0.26 | 0.59 | 6.45E-06 | 0.91 | 2.17E-01 | 0.79 | 3.55E-04 |
| 11q25 | rs12364540 | IGSF9B | T>C | 0.51 | 0.46 | 1.36 | 1.09E-03 | 1.2 | 9.07E-03 | 1.25 | 5.46E-05 |
| 12p11.22 | rs7977334 | TMTC1 | T>C | 0.12 | 0.08 | 1.89 | 5.90E-05 | 1.2 | 1.05E-01 | 1.4 | 2.18E-04 |
| 12p13.31 | rs11053534 | CD163L1 | T>C | 0.08 | 0.05 | 1.32 | 1.31E-01 | 1.86 | 1.61E-05 | 1.64 | 1.24E-05 |
| 12q13.12 | rs11168830 | MLL2 | G>A | 0.27 | 0.24 | 1.30 | 1.15E-02 | 1.27 | 2.40E-03 | 1.28 | 7.86E-05 |
| 12q23.1 | rs6538797 | NEDD1 | C>T | 0.31 | 0.26 | 1.28 | 1.75E-02 | 1.26 | 1.73E-03 | 1.27 | 8.51E-05 |
| 12q24.13 | rs7313883 | LHX5 | A>G | 0.43 | 0.50 | 0.80 | 1.42E-02 | 0.81 | 1.49E-03 | 0.8 | 6.03E-05 |
| 13q12.11 | rs3129595 | FGF9/LOC646201 | C>T | 0.12 | 0.10 | 1.26 | 1.17E-01 | 1.51 | 1.87E-04 | 1.41 | 8.31E-05 |
| 13q21.32 | rs11148740 | LOC730236/KLHL1 | C>T | 0.17 | 0.21 | 0.89 | 2.76E-01 | 0.71 | 7.88E-05 | 0.77 | 1.60E-04 |
| 14q24.2 | rs7149261 | SYNJ2BP | T>G | 0.09 | 0.07 | 1.46 | 2.51E-02 | 1.61 | 3.40E-04 | 1.55 | 2.66E-05 |
| 14q32.2 | rs234601 | PAPOLA | T>C | 0.48 | 0.44 | 1.23 | 2.03E-02 | 1.26 | 7.47E-04 | 1.25 | 4.31E-05 |
| 16p13.2 | rs7197658 | A2BP1/TMEM114 | T>G | 0.36 | 0.40 | 0.67 | 5.92E-05 | 0.91 | 1.55E-01 | 0.82 | 4.99E-04 |
| 16q21 | rs4151117 | CX3CL1 | T>G | 0.32 | 0.38 | 0.68 | 4.70E-05 | 0.91 | 1.66E-01 | 0.82 | 3.37E-04 |
| 17q21.31 | rs7215223 | ARL4D | C>T | 0.34 | 0.40 | 0.79 | 1.18E-02 | 0.8 | 1.69E-03 | 0.8 | 5.75E-05 |
| 19q13.42 | rs17305346 | LILRB2 | G>A | 0.14 | 0.16 | 0.72 | 3.14E-02 | 0.73 | 4.46E-04 | 0.73 | 3.83E-05 |
| 20q12 | rs3092194 | CHD6 | G>A | 0.06 | 0.08 | 0.74 | 1.12E-01 | 0.56 | 8.20E-06 | 0.61 | 4.41E-06 |
| 20q13.32 | rs7262054 | LOC100129869 | C>T | 0.20 | 0.22 | 0.7 | 2.39E-03 | 0.8 | 6.09E-03 | 0.77 | 6.84E-05 |
| 21q21.3 | rs455804 | GRIK1 | C>A | 0.30 | 0.35 | 0.92 | 3.69E-01 | 0.75 | 6.64E-05 | 0.81 | 1.65E-04 |
| 21q22.11 | rs743417 | FLJ46020 | T>C | 0.40 | 0.34 | 1.47 | 9.63E-05 | 1.18 | 1.67E-02 | 1.27 | 2.51E-05 |

^a^ Major>minor alleles; ^b^ MAF, Minor allele-frequency. ^c^ OR_c, *P*_c : OR and *P* values for the Central study samples in GWAS scan adjusted by the first principal component; OR_s, *P*_s : OR and *P* values for the Southern study samples in GWAS scan adjusted by the first principal component; ^d^ OR_all, *P*_all: OR and *P* values for combining Central and Southern studies in GWAS scan by joint analysis.

**B:**  Associations of 39 SNPs in GWAS scan and validations

| **Chr.^a^** | **SNP** | **Study** | **Cases^b^** | **Controls^b^** | **MAF^c^** | | **OR_add_^d^** | **95% CI^d^** | **P_add_^d^** |
| --- | --- | --- | --- | --- | --- | --- | --- | --- | --- |
|  |  |  |  |  | **Cases** | **Controls** |  |  |  |
| 1p36.32 | rs7518471 | GWAS Scan | 13/211/1314 | 8/153/1304 | 0.08 | 0.06 | 1.48 | (1.19-1.84) | 5.25E-04 |
| C/T |  | Validation 1 | 12/280/1796 | 10/326/1838 | 0.07 | 0.08 | 0.90 | (0.77-1.06) | 2.06E-01 |
| 1q24.2 | rs7512378 | GWAS Scan | 97/567/868 | 69/445/948 | 0.25 | 0.20 | 1.32 | (1.16-1.50) | 3.50E-05 |
| A/G |  | Validation 1 | 93/706/1296 | 109/792/1299 | 0.21 | 0.23 | 0.90 | (0.82-1.00) | 5.74E-02 |
| 2q22.1 | rs7424161 | GWAS Scan | 80/542/915 | 113/599/751 | 0.23 | 0.28 | 0.76 | (0.67-0.86) | 2.69E-05 |
| C/T |  | Validation 1 | 129/798/1111 | 134/820/1209 | 0.26 | 0.25 | 1.04 | (0.94-1.15) | 4.25E-01 |
| 2q22.3 | rs1317530 | GWAS Scan | 267/778/493 | 204/696/565 | 0.43 | 0.38 | 1.28 | (1.15-1.44) | 1.80E-05 |
| C/T |  | Validation 1 | 377/1015/719 | 359/1026/753 | 0.42 | 0.41 | 1.04 | (0.96-1.14) | 3.46E-01 |
| 2q23.1 | rs6721330 | GWAS Scan | 84/562/890 | 50/482/932 | 0.24 | 0.20 | 1.34 | (1.17-1.54) | 2.23E-05 |
| C/A |  | Validation 1 | 95/735/1244 | 106/743/1307 | 0.22 | 0.22 | 1.00 | (0.90-1.11) | 9.55E-01 |
| 3q13.13 | rs10933971 | GWAS Scan | 318/802/413 | 260/683/512 | 0.47 | 0.41 | 1.27 | (1.14-1.42) | 2.78E-05 |
| C/T |  | Validation 1 | 401/1046/643 | 436/1097/645 | 0.44 | 0.45 | 0.96 | (0.88-1.05) | 3.96E-01 |
| 4p14 | rs907314 | GWAS Scan | 30/367/1139 | 47/421/997 | 0.14 | 0.18 | 0.73 | (0.63-0.85) | 6.08E-05 |
| C/T |  | Validation 1 | 43/519/1505 | 38/559/1565 | 0.15 | 0.15 | 0.99 | (0.88-1.12) | 8.76E-01 |
| 5q14.1 | rs16877319 | GWAS Scan | 7/244/1287 | 5/161/1298 | 0.08 | 0.06 | 1.57 | (1.26-1.96) | 5.06E-05 |
| T/C |  | Validation 1 | 39/259/1770 | 17/275/1874 | 0.08 | 0.07 | 1.13 | (0.97-1.32) | 1.08E-01 |
| 5q35.2 | rs2964057 | GWAS Scan | 82/586/870 | 62/467/934 | 0.24 | 0.20 | 1.25 | (1.09-1.43) | 1.21E-03 |
| A/G |  | Validation 1 | 95/672/1342 | 108/674/1405 | 0.20 | 0.20 | 1.01 | (0.91-1.12) | 8.50E-01 |
| **6p21.32** | **rs9272105** | **GWAS Scan** | **477/685/373** | **320/699/440** | **0.53** | **0.46** | **1.31** | **(1.18-1.46)** | **8.86E-07** |
| **G/A** |  | **Validation 1** | **580/976/556** | **436/1118/653** | **0.51** | **0.45** | **1.23** | **(1.13-1.34)** | **1.04E-06** |
|  |  | **Validation 2** | **278/420/242** | **260/770/459** | **0.52** | **0.43** | **1.41** | **(1.25-1.58)** | **7.63E-09** |
|  |  | **Combined All** | **1335/2081/1171** | **1016/2587/1552** | **0.52** | **0.45** | **1.30** | **(1.23-1.38)** | **1.13E-19** |
| 6p22.3 | rs7739131 | GWAS Scan | 238/732/567 | 168/666/627 | 0.39 | 0.34 | 1.27 | (1.13-1.42) | 6.59E-05 |
| C/T |  | Validation 1 | 249/971/858 | 285/973/910 | 0.35 | 0.36 | 0.99 | (0.90-1.08) | 8.08E-01 |
| 6q14.1 | rs9341799 | GWAS Scan | 237/698/600 | 270/711/482 | 0.38 | 0.43 | 0.80 | (0.71-0.89) | 7.26E-05 |
| C/T |  | Validation 1 | 350/939/823 | 408/977/823 | 0.39 | 0.41 | 0.93 | (0.86-1.01) | 9.93E-02 |
| 7p21.3 | rs2189638 | GWAS Scan | 270/720/547 | 177/653/629 | 0.41 | 0.35 | 1.24 | (1.11-1.39) | 2.31E-04 |
| A/G |  | Validation 1 | 252/938/912 | 235/960/909 | 0.34 | 0.34 | 1.01 | (0.92-1.10) | 8.72E-01 |
| 7q32.3 | rs1465387 | GWAS Scan | 124/601/809 | 92/499/874 | 0.28 | 0.23 | 1.27 | (1.12-1.44) | 1.60E-04 |
| A/G |  | Validation 1 | 141/843/1108 | 152/804/1235 | 0.27 | 0.25 | 1.08 | (0.98-1.20) | 1.02E-01 |
| 9p23 | rs1822387 | GWAS Scan | 281/779/478 | 245/685/534 | 0.44 | 0.40 | 1.23 | (1.10-1.38) | 3.08E-04 |
| C/T |  | Validation 1 | 408/997/644 | 443/1075/642 | 0.44 | 0.45 | 0.96 | (0.88-1.05) | 3.37E-01 |
| 9q21.33 | rs7036455 | GWAS Scan | 285/772/481 | 328/747/390 | 0.44 | 0.48 | 0.81 | (0.72-0.90) | 1.94E-04 |
| T/C |  | Validation 1 | 403/1057/626 | 444/1081/648 | 0.45 | 0.45 | 0.97 | (0.89-1.06) | 5.53E-01 |
| 10p11.22 | rs2666261 | GWAS Scan | 82/585/869 | 58/469/938 | 0.24 | 0.20 | 1.35 | (1.18-1.54) | 1.73E-05 |
| C/T |  | Validation 1 | 91/691/1308 | 88/687/1397 | 0.21 | 0.20 | 1.06 | (0.96-1.18) | 2.64E-01 |
| 10q23.2 | rs12782065 | GWAS Scan | 15/277/1245 | 27/322/1115 | 0.10 | 0.13 | 0.72 | (0.61-0.86) | 2.17E-04 |
| C/T |  | Validation 1 | 30/399/1683 | 27/438/1682 | 0.11 | 0.11 | 0.95 | (0.83-1.09) | 4.56E-01 |
| 11p15.1 | rs3825023 | GWAS Scan | 32/401/1103 | 12/283/1168 | 0.15 | 0.10 | 1.50 | (1.27-1.78) | 2.62E-06 |
| C/T |  | Validation 1 | 25/397/1652 | 25/394/1782 | 0.11 | 0.10 | 1.07 | (0.93-1.23) | 3.25E-01 |
| 11q13.1 | rs10160758 | GWAS Scan | 84/575/879 | 60/465/940 | 0.24 | 0.20 | 1.34 | (1.17-1.53) | 1.73E-05 |
| T/C |  | Validation 1 | 108/734/1224 | 126/717/1332 | 0.23 | 0.22 | 1.04 | (0.94-1.15) | 4.19E-01 |
| 11q13.3 | rs10896464 | GWAS Scan | 81/573/883 | 95/562/807 | 0.24 | 0.26 | 0.77 | (0.67-0.88) | 8.12E-05 |
| C/T |  | Validation 1 | 75/662/1358 | 94/670/1420 | 0.19 | 0.20 | 0.99 | (0.89-1.11) | 9.25E-01 |
| 11q25 | rs12364540 | GWAS Scan | 388/777/369 | 298/750/416 | 0.51 | 0.46 | 1.24 | (1.11-1.38) | 1.96E-04 |
| T/C |  | Validation 1 | 511/983/588 | 491/1071/606 | 0.48 | 0.47 | 1.04 | (0.95-1.13) | 4.15E-01 |
| 12p11.22 | rs7977334 | GWAS Scan | 23/319/1175 | 13/219/1214 | 0.12 | 0.08 | 1.37 | (1.14-1.65) | 8.60E-04 |
| T/C |  | Validation 1 | 21/357/1726 | 24/358/1797 | 0.09 | 0.09 | 1.02 | (0.89-1.18) | 7.48E-01 |
| 12p13.31 | rs11053534 | GWAS Scan | 10/221/1303 | 3/150/1312 | 0.08 | 0.05 | 1.66 | (1.32-2.08) | 1.58E-05 |
| T/C |  | Validation 1 | 12/265/1835 | 13/246/1902 | 0.07 | 0.06 | 1.10 | (0.92-1.30) | 2.93E-01 |
| 12q13.12 | rs11168830 | GWAS Scan | 118/601/819 | 86/529/848 | 0.27 | 0.24 | 1.30 | (1.14-1.47) | 6.06E-05 |
| G/A |  | Validation 1 | 194/848/1050 | 187/919/1068 | 0.30 | 0.30 | 0.99 | (0.90-1.09) | 8.56E-01 |
| 12q23.1 | rs6538797 | GWAS Scan | 155/640/743 | 107/556/802 | 0.31 | 0.26 | 1.26 | (1.12-1.42) | 1.63E-04 |
| C/T |  | Validation 1 | 158/810/1119 | 189/888/1094 | 0.27 | 0.29 | 0.90 | (0.82-0.99) | 2.48E-02 |
| 12q24.13 | rs7313883 | GWAS Scan | 288/741/508 | 376/708/380 | 0.43 | 0.50 | 0.81 | (0.73-0.91) | 2.50E-04 |
| A/G |  | Validation 1 | 495/1023/557 | 509/1102/558 | 0.49 | 0.49 | 0.98 | (0.90-1.07) | 6.54E-01 |
| 13q12.11 | rs3129595 | GWAS Scan | 30/314/1193 | 15/252/1198 | 0.12 | 0.10 | 1.46 | (1.22-1.74) | 2.72E-05 |
| C/T |  | Validation 1 | 26/419/1645 | 27/456/1692 | 0.11 | 0.12 | 0.95 | (0.83-1.09) | 4.51E-01 |
| **13q21.32** | **rs11148740** | **GWAS Scan** | **42/453/1043** | **77/474/913** | **0.17** | **0.21** | **0.78** | **(0.68-0.90)** | **4.33E-04** |
| **C/T** |  | **Validation 1** | **70/644/1335** | **103/704/1364** | **0.19** | **0.21** | **0.89** | **(0.80-0.99)** | **3.15E-02** |
|  |  | **Validation 2** | **39/311/670** | **57/448/972** | **0.19** | **0.19** | **0.99** | **(0.86-1.15)** | **9.28E-01** |
| 14q24.2 | rs7149261 | GWAS Scan | 13/243/1278 | 3/186/1273 | 0.09 | 0.07 | 1.47 | (1.19-1.81) | 3.73E-04 |
| T/G |  | Validation 1 | 15/321/1744 | 15/327/1830 | 0.08 | 0.08 | 1.02 | (0.88-1.20) | 7.70E-01 |
| 14q32.2 | rs234601 | GWAS Scan | 368/752/417 | 295/703/467 | 0.48 | 0.44 | 1.21 | (1.08-1.35) | 7.81E-04 |
| T/C |  | Validation 1 | 479/1027/577 | 444/1116/603 | 0.48 | 0.46 | 1.06 | (0.97-1.16) | 1.82E-01 |
| 16p13.2 | rs7197658 | GWAS Scan | 202/699/637 | 218/727/520 | 0.36 | 0.40 | 0.83 | (0.74-0.94) | 1.96E-03 |
| T/G |  | Validation 1 | 287/949/845 | 314/1009/852 | 0.37 | 0.38 | 0.96 | (0.88-1.04) | 3.17E-01 |
| 16q21 | rs4151117 | GWAS Scan | 169/638/730 | 218/664/582 | 0.32 | 0.38 | 0.84 | (0.75-0.94) | 2.14E-03 |
| T/G |  | Validation 1 | 66/1905/141 | 95/1922/190 | 0.48 | 0.48 | 1.05 | (0.88-1.26) | 5.92E-01 |
| 17q21.31 | rs7215223 | GWAS Scan | 183/670/685 | 244/696/524 | 0.34 | 0.40 | 0.79 | (0.71-0.89) | 7.12E-05 |
| C/T |  | Validation 1 | 359/1014/709 | 403/1042/724 | 0.42 | 0.43 | 0.96 | (0.88-1.04) | 3.16E-01 |
| 19q13.42 | rs17305346 | GWAS Scan | 28/364/1145 | 45/375/1043 | 0.14 | 0.16 | 0.71 | (0.61-0.83) | 1.71E-05 |
| G/A |  | Validation 1 | 20/341/1732 | 20/349/1831 | 0.09 | 0.09 | 1.03 | (0.89-1.19) | 7.29E-01 |
| 20q12 | rs3092194 | GWAS Scan | 7/171/1359 | 10/228/1225 | 0.06 | 0.08 | 0.63 | (0.51-0.78) | 2.98E-05 |
| G/A |  | Validation 1 | 4/230/1852 | 6/246/1923 | 0.06 | 0.06 | 0.97 | (0.80-1.16) | 7.07E-01 |
| 20q13.32 | rs7262054 | GWAS Scan | 68/466/1004 | 72/503/888 | 0.20 | 0.22 | 0.76 | (0.67-0.87) | 9.54E-05 |
| C/T |  | Validation 1 | 63/623/1376 | 70/615/1483 | 0.18 | 0.17 | 1.05 | (0.94-1.17) | 4.04E-01 |
| **21q21.3** | **rs455804** | **GWAS Scan** | **157/612/769** | **182/655/628** | **0.30** | **0.35** | **0.79** | **(0.71-0.89)** | **1.02E-04** |
| **C/A** |  | **Validation 1** | **201/888/1021** | **262/962/976** | **0.31** | **0.34** | **0.86** | **(0.79-0.94)** | **1.15E-03** |
|  |  | **Validation 2** | **89/426/506** | **154/689/648** | **0.30** | **0.33** | **0.83** | **(0.74-0.94)** | **3.63E-03** |
|  |  | **Combined All** | **447/1926/2296** | **598/2306/2252** | **0.30** | **0.34** | **0.84** | **(0.79-0.89)** | **1.86E-08** |
| 21q22.11 | rs743417 | GWAS Scan | 249/744/544 | 170/661/634 | 0.40 | 0.34 | 1.25 | (1.12-1.40) | 1.29E-04 |
| T/C |  | Validation 1 | 260/1020/808 | 296/967/913 | 0.37 | 0.36 | 1.05 | (0.96-1.14) | 3.19E-01 |

^a^ Major/minor alleles; ^b^ Variant homozygote/Heterozygote/Wild type homozygote; ^c^ MAF, Minor allele frequency; ^d^ Adjusted by the first principal component, sex, age, smoking and alcohol consumption;.
