# Supplementary material for: GWAS Identifies Novel Susceptibility Loci on 6p21.32 and 21q21.3 for Hepatocellular Carcinoma in Chronic Hepatitis B Virus Carriers
Source: PLoS Genet. 2012 Jul 12;8(7):e1002791. doi: 10.1371/journal.pgen.1002791 (PMC3395595; doi:10.1371/journal.pgen.1002791)
Supplement: Table S4 — Adjusted and stratified analyses of the 2 validated SNPs. (DOCX) [file pgen.1002791.s010.docx]

**Table S4** Adjusted and stratified analyses of the 2 validated SNPs

A: Adjusted association analysis by age, gender, drinking and smoking

| **Results** | **6p21.32: rs9272105** | | **21q21.3: rs455804** | |
| --- | --- | --- | --- | --- |
|  | **Joint Analysis 1^a^** | **Joint Analysis 2^b^** | **Joint Analysis 1^a^** | **Joint Analysis 2^b^** |
| **OR (95% CI)** | 1.28(1.22-1.35) | 1.30(1.23-1.36) | 0.84(0.80-0.89) | 0.84 (0.79-0.89) |
| ***P*** | 5.24E-22 | 3.32E-23 | 5.24E-10 | 4.43E-10 |

^a^ Joint analysis 1: Joint analysis of GWAS and replication studies adjusted by the first principal component;

^b^ Joint analysis 2: Joint analysis of GWAS and replication studies adjusted by the first principal component, age, gender, drinking, and smoking.

B: Stratification analysis by age, gender, drinking and smoking

| **Variables** | **6p21.32: rs9272105** | | | **21q21.3: rs455804** | | |
| --- | --- | --- | --- | --- | --- | --- |
|  | **OR (95% CI)^a^** | ***P^a^*** | ***P^b^*** | **OR (95% CI)^a^** | ***P^a^*** | ***P^b^*** |
| **Age** |  |  | 0.560 |  |  | 0.287 |
| **<50** | 1.31(1.21-1.41) | 2.87E-12 |  | 0.87(0.80-0.94) | 7.02E-04 |  |
| **≥50** | 1.27(1.18-1.36) | 1.89E-11 |  | 0.82(0.76-0.88) | 1.10E-07 |  |
| **Gender** |  |  | 0.494 |  |  | 0.646 |
| **Male** | 1.31(1.24-1.38) | 1.00E-20 |  | 0.84(0.79-0.89) | 5.76E-09 |  |
| **Female** | 1.25(1.11-1.42) | 3.27E-04 |  | 0.87(0.76-1.00) | 4.39E-02 |  |
| **Drinking status** |  |  | 0.467 |  |  | 0.833 |
| **No** | 1.27(1.19-1.35) | 7.02E-13 |  | 0.84(0.79-0.90) | 1.97E-06 |  |
| **Yes** | 1.32(1.22-1.44) | 5.67E-11 |  | 0.83(0.76-0.91) | 2.88E-05 |  |
| **Smoking status** |  |  | 0.004 |  |  | 0.538 |
| **No** | 1.38(1.28-1.48) | 3.36E-18 |  | 0.86(0.79-0.93) | 9.43E-05 |  |
| **Yes** | 1.19(1.11-1.28) | 1.90E-06 |  | 0.83(0.77-0.90) | 2.01E-06 |  |

^a^ Joint analysis of GWAS and replication studies adjusted by the first principal component. ^b^ *P* for heterogeneity tests based on Cochrane’s Q test.
