# Supplementary material for: GWAS Identifies Novel Susceptibility Loci on 6p21.32 and 21q21.3 for Hepatocellular Carcinoma in Chronic Hepatitis B Virus Carriers
Source: PLoS Genet. 2012 Jul 12;8(7):e1002791. doi: 10.1371/journal.pgen.1002791 (PMC3395595; doi:10.1371/journal.pgen.1002791)
Supplement: Table S5 — Results of all the HLA alleles that have been successfully imputed after quality controls. (DOCX) [file pgen.1002791.s011.docx]

**Table S5** Results of all the HLA alleles that have been successfully imputed after quality controls.

| **CHR** | **HLA-allele** | **Position** | **Minor allele** | **Major allele** | **MAF ^a^** | | **OR_s ^b^** | ***P*_s ^b^** | **OR_c ^c^** | ***P*_c ^c^** | **OR_all ^d^** | ***P*_all ^d^** |
| --- | --- | --- | --- | --- | --- | --- | --- | --- | --- | --- | --- | --- |
|  |  |  |  |  | **Case** | **Control** |  |  |  |  |  |  |
| 6 | HLA_DRB1_0405 | 32660042 | G | A | 0.05 | 0.08 | 0.68 | 2.79E-03 | 0.71 | 9.33E-02 | 0.69 | 6.18E-04 |
| 6 | HLA_DQA1_0301 | 32716284 | G | A | 0.30 | 0.35 | 0.9 | 1.62E-01 | 0.76 | 4.22E-03 | 0.85 | 4.39E-03 |
| 6 | HLA_DQB1_0401 | 32739039 | G | A | 0.04 | 0.06 | 0.67 | 8.28E-03 | 0.78 | 2.26E-01 | 0.71 | 4.49E-03 |
| 6 | HLA_DQA1_0601 | 32716284 | G | A | 0.15 | 0.12 | 1.24 | 2.64E-02 | 1.24 | 1.29E-01 | 1.24 | 7.12E-03 |
| 6 | HLA_DRB1_0901 | 32660042 | G | A | 0.18 | 0.22 | 0.93 | 3.77E-01 | 0.73 | 4.15E-03 | 0.85 | 1.46E-02 |
| 6 | HLA_A_3001 | 30019970 | G | A | 0.03 | 0.03 | 1.45 | 1.46E-01 | 1.25 | 3.20E-01 | 1.33 | 8.87E-02 |
| 6 | HLA_A_3101 | 30019970 | G | A | 0.03 | 0.03 | 1.12 | 6.67E-01 | 1.32 | 1.51E-01 | 1.24 | 1.59E-01 |
| 6 | HLA_C_1502 | 31346171 | G | A | 0.04 | 0.03 | 1.12 | 5.37E-01 | 1.48 | 1.24E-01 | 1.23 | 1.60E-01 |
| 6 | HLA_C_0304 | 31346171 | G | A | 0.09 | 0.09 | 0.86 | 1.67E-01 | 0.96 | 7.93E-01 | 0.88 | 1.92E-01 |
| 6 | HLA_B_5101 | 31431272 | G | A | 0.05 | 0.06 | 1.04 | 7.83E-01 | 0.53 | 6.36E-03 | 0.86 | 2.06E-01 |
| 6 | HLA_B_1302 | 31431272 | G | A | 0.03 | 0.03 | 1.21 | 4.08E-01 | 1.20 | 4.20E-01 | 1.20 | 2.48E-01 |
| 6 | HLA_DQA1_0103 | 32716284 | G | A | 0.12 | 0.12 | 1.09 | 3.83E-01 | 1.10 | 5.07E-01 | 1.10 | 2.74E-01 |
| 6 | HLA_DRB1_0301 | 32660042 | G | A | 0.07 | 0.05 | 1.11 | 4.80E-01 | 1.17 | 4.05E-01 | 1.13 | 2.87E-01 |
| 6 | HLA_C_0102 | 31346171 | G | A | 0.25 | 0.26 | 0.94 | 3.85E-01 | 0.94 | 5.79E-01 | 0.94 | 3.04E-01 |
| 6 | HLA_DQA1_0201 | 32716284 | G | A | 0.03 | 0.03 | 1.23 | 4.32E-01 | 1.14 | 5.07E-01 | 1.17 | 3.18E-01 |
| 6 | HLA_A_2402 | 30019970 | G | A | 0.16 | 0.17 | 0.99 | 9.27E-01 | 0.83 | 1.34E-01 | 0.93 | 3.22E-01 |
| 6 | HLA_B_1301 | 31431272 | G | A | 0.06 | 0.06 | 0.85 | 2.15E-01 | 1.05 | 8.13E-01 | 0.90 | 3.47E-01 |
| 6 | HLA_DRB1_0701 | 32660042 | G | A | 0.04 | 0.05 | 1.28 | 2.13E-01 | 1.03 | 8.75E-01 | 1.13 | 3.55E-01 |
| 6 | HLA_C_0801 | 31346171 | G | A | 0.10 | 0.08 | 1.3 | 2.44E-02 | 0.76 | 9.55E-02 | 1.09 | 3.77E-01 |
| 6 | HLA_B_3501 | 31431272 | G | A | 0.02 | 0.03 | 0.74 | 2.51E-01 | 0.96 | 8.57E-01 | 0.86 | 3.79E-01 |
| 6 | HLA_C_0602 | 31346171 | G | A | 0.04 | 0.05 | 1.23 | 3.14E-01 | 1.05 | 7.94E-01 | 1.13 | 3.91E-01 |
| 6 | HLA_A_0206 | 30019970 | G | A | 0.04 | 0.04 | 1 | 9.95E-01 | 0.80 | 2.44E-01 | 0.89 | 4.03E-01 |
| 6 | HLA_A_0207 | 30019970 | G | A | 0.13 | 0.13 | 0.92 | 3.98E-01 | 0.97 | 8.41E-01 | 0.94 | 4.06E-01 |
| 6 | HLA_A_3303 | 30019970 | G | A | 0.11 | 0.09 | 1.1 | 4.18E-01 | 0.97 | 8.37E-01 | 1.05 | 5.76E-01 |
| 6 | HLA_B_4601 | 31431272 | G | A | 0.18 | 0.18 | 0.94 | 4.80E-01 | 1.01 | 9.40E-01 | 0.96 | 5.85E-01 |
| 6 | HLA_A_0201 | 30019970 | G | A | 0.15 | 0.15 | 0.92 | 3.91E-01 | 1.08 | 5.47E-01 | 0.98 | 7.41E-01 |
| 6 | HLA_B_4002 | 31431272 | G | A | 0.05 | 0.05 | 0.91 | 5.61E-01 | 1.04 | 8.46E-01 | 0.96 | 7.43E-01 |
| 6 | HLA_DQB1_0201 | 32739039 | G | A | 0.15 | 0.14 | 0.94 | 5.49E-01 | 1.20 | 1.67E-01 | 1.03 | 7.45E-01 |
| 6 | HLA_A_1101 | 30019970 | G | A | 0.30 | 0.29 | 1.01 | 8.78E-01 | 1.03 | 7.72E-01 | 1.02 | 7.74E-01 |
| 6 | HLA_C_0303 | 31346171 | G | A | 0.07 | 0.08 | 0.85 | 2.36E-01 | 1.16 | 3.42E-01 | 0.97 | 7.97E-01 |
| 6 | HLA_B_4001 | 31431272 | G | A | 0.14 | 0.14 | 0.96 | 6.40E-01 | 1.23 | 1.95E-01 | 1.02 | 8.08E-01 |
| 6 | HLA_C_0302 | 31346171 | G | A | 0.08 | 0.07 | 1.06 | 6.63E-01 | 0.96 | 8.23E-01 | 1.02 | 8.25E-01 |
| 6 | HLA_B_4801 | 31431272 | G | A | 0.01 | 0.01 | 1.33 | 3.64E-01 | 0.76 | 4.51E-01 | 1.05 | 8.45E-01 |
| 6 | HLA_DQB1_0601 | 32739039 | G | A | 0.12 | 0.11 | 1.06 | 6.16E-01 | 0.95 | 7.16E-01 | 1.02 | 8.48E-01 |
| 6 | HLA_B_5401 | 31431272 | G | A | 0.08 | 0.08 | 0.92 | 5.18E-01 | 1.11 | 5.47E-01 | 0.98 | 8.54E-01 |
| 6 | HLA_C_0702 | 31346171 | G | A | 0.20 | 0.19 | 1 | 9.61E-01 | 1.04 | 7.71E-01 | 1.01 | 9.02E-01 |
| 6 | HLA_B_5801 | 31431272 | G | A | 0.08 | 0.07 | 1.02 | 8.82E-01 | 0.99 | 9.49E-01 | 1.01 | 9.35E-01 |

^a^ MAF:Minor Allele Frequency; ^b^OR_s, *P*_s: OR and *P* values for the Southern study samples in GWAS scan adjusted by the first principal component; ^c^OR_c, *P*_c : OR and *P* values for the Central study samples in GWAS scan adjusted by the first principal component; ^d^ OR_all, *P*_all: OR and *P* values for combining Central and Southern studies in GWAS scan by joint analysis.
