# Supplementary material for: GWAS Identifies Novel Susceptibility Loci on 6p21.32 and 21q21.3 for Hepatocellular Carcinoma in Chronic Hepatitis B Virus Carriers
Source: PLoS Genet. 2012 Jul 12;8(7):e1002791. doi: 10.1371/journal.pgen.1002791 (PMC3395595; doi:10.1371/journal.pgen.1002791)
Supplement: Table S6 — The haplotype analysis of the two DRB1 alleles and rs9272105. (DOCX) [file pgen.1002791.s012.docx]

**Table S6** The haplotype analysis of the two DRB1 alleles and rs9272105

| **CHR** | **Haplotype** | **Southern cohort** | | **Central cohort** | | **Joint analysis** | |
| --- | --- | --- | --- | --- | --- | --- | --- |
|  |  | **OR (95% CI)** | ***P*** | **OR (95% CI)** | ***P*** | **OR (95% CI)** | ***P*** |
| 6 | HLA_DRB1_0405(A)\|HLA_DRB1_0901(A)\|rs9272105(A) | 1.30 (1.15-1.46) | 1.77E-05 | 1.40 (1.17-1.68) | 2.98E-04 | 1.32 (1.19-1.46) | 2.06E-07 |
| 6 | HLA_DRB1_0405(A)\|HLA_DRB1_0901(G)\|rs9272105(G) | 0.86 (0.74-1.00) | 4.67E-02 | 0.74 (0.59-0.91) | 5.10E-03 | 0.85 (0.75-0.97) | 1.30E-02 |
| 6 | HLA_DRB1_0405(G)\|HLA_DRB1_0901(A)\|rs9272105(G) | 0.61 (0.48-0.77) | 4.69E-05 | 0.72 (0.48-1.08) | 1.13E-01 | 0.68 (0.55-0.85) | 6.48E-04 |
| 6 | HLA_DRB1_0405(A)\|HLA_DRB1_0901(A)\|rs9272105(G) | 0.94 (0.82-1.09) | 4.04E-01 | 0.94 (0.76-1.15) | 5.33E-01 | 0.89 (0.78-1.01) | 6.08E-02 |

For HLA alleles: “G” means present, and "A" means absent
