# Supplementary material for: GWAS Identifies Novel Susceptibility Loci on 6p21.32 and 21q21.3 for Hepatocellular Carcinoma in Chronic Hepatitis B Virus Carriers
Source: PLoS Genet. 2012 Jul 12;8(7):e1002791. doi: 10.1371/journal.pgen.1002791 (PMC3395595; doi:10.1371/journal.pgen.1002791)
Supplement: Table S7 — A: Association of two SNPs in the subjects with asymptomatic and symptomatic HBV infection from the southern Chinese cohort. B: Association of rs9272105 and rs455804 in asymptomatic HBV carriers and HBV natural clearance samples from central China. (DOCX) [file pgen.1002791.s013.docx]

**Table S7**

A: Association of two SNPs in the subjects with asymptomatic and symptomatic HBV infection from the southern Chinese cohort

| **CHR** | **SNP** | **BP** | **Allele** | **Test Allele** | **TAF in asymptomatic HBV carriers (408)** | **TAF in chronic hepatitis B patients (521)** | **TAF in HBV-induced HCC patients (1058)** | **Chronic hepatitis B patients vs. asymptomatic HBV carriers** | | **HBV-induced HCC vs. chronic hepatitis B patients** | |
| --- | --- | --- | --- | --- | --- | --- | --- | --- | --- | --- | --- |
|  |  |  |  |  |  |  |  | **OR (95% CI)** | ***P*** | **OR (95% CI)** | ***P*** |
| 6 | rs9272105 | 32707977 | G/A | A | 0.51 | 0.45 | 0.55 | 0.80 (0.67-0.96) | 1.67E-02 | 1.40 (1.21-1.61) | 3.95E-06 |
| 21 | rs455804 | 30068040 | C/A | A | 0.34 | 0.36 | 0.29 | 1.08 (0.89-1.31) | 4.24E-01 | 0.74 (0.64-0.87) | 1.72E-04 |

B: Association of rs9272105 and rs455804 in asymptomatic HBV carriers and HBV natural clearance samples from central China.

| **CHR** | **SNP** | **BP** | **Allele** | **Test Allele** | **TAF in asymptomatic HBV carriers (4183)** | **TAF in HBV natural clearance subjects**  **(1344)** | **Asymptomatic HBV carriers vs. HBV natural clearance subjects** | |
| --- | --- | --- | --- | --- | --- | --- | --- | --- |
|  |  |  |  |  |  |  | **OR (95% CI)** | ***P*** |
| 6 | rs9272105 | 32707977 | G/A | A | 0.44 | 0.47 | 0.88 (0.80-0.96) | 3.78E-03 |
| 21 | rs455804 | 30068040 | C/A | A | 0.34 | 0.32 | 1.09 (0.99-1.20) | 7.27E-02 |

TAF: test allele frequency
