# Supplementary material for: GWAS Identifies Novel Susceptibility Loci on 6p21.32 and 21q21.3 for Hepatocellular Carcinoma in Chronic Hepatitis B Virus Carriers
Source: PLoS Genet. 2012 Jul 12;8(7):e1002791. doi: 10.1371/journal.pgen.1002791 (PMC3395595; doi:10.1371/journal.pgen.1002791)
Supplement: Table S8 — Association results of rs17401966, rs2596542, rs1012068, and rs9275572 in the GWAS samples. (DOCX) [file pgen.1002791.s014.docx]

**Table S8** Association results of rs17401966, rs2596542, rs1012068 and rs9275572 in the GWAS samples

| **SNP** | **GWAS Samples** | **MAF** ^b^ | | **OR (95% CI)** | ***P*** |
| --- | --- | --- | --- | --- | --- |
|  |  | **Cases** | **Controls** |  |  |
| **1p36.22: rs17401966** | **Southern** | 0.27 | 0.28 | 0.89 (0.77-1.04) | 1.49E-01 |
| **A/G ^a^** | **Central** | 0.27 | 0.29 | 0.91 (0.74-1.12) | 3.75E-01 |
|  | **Joint GWAS** | 0.27 | 0.29 | 0.90 (0.80-1.02) | 9.13E-02 |
| **6p21.32: rs9275572** | **Southern** | 0.21 | 0.22 | 0.92 (0.79-1.08) | 3.30E-01 |
| **G/A ^a^** | **Central** | 0.22 | 0.24 | 0.93 (0.75-1.15) | 4.97E-01 |
|  | **Joint GWAS** | 0.21 | 0.22 | 0.93 (0.81-1.05) | 2.35E-01 |
| **6p21.33: rs2596542** | **Southern** | 0.29 | 0.27 | 1.01 (0.87-1.17) | 8.97E-01 |
| **C/T ^a^** | **Central** | 0.30 | 0.27 | 1.15 (0.94-1.40) | 1.77E-01 |
|  | **Joint GWAS** | 0.29 | 0.27 | 1.06 (0.94-1.19) | 3.64E-01 |
| **22q12.3: rs1012068** | **Southern** | 0.24 | 0.26 | 1.01 (0.87-1.17) | 9.18E-01 |
| **T/G ^a^** | **Central** | 0.25 | 0.23 | 1.16 (0.94-1.44) | 1.66E-01 |
|  | **Joint GWAS** | 0.25 | 0.25 | 1.06 (0.93-1.20) | 3.74E-01 |

^a^ Major allele/minor allele; ^b^ MAF: minor allele frequency.
