# Supplementary material for: GWAS Identifies Novel Susceptibility Loci on 6p21.32 and 21q21.3 for Hepatocellular Carcinoma in Chronic Hepatitis B Virus Carriers
Source: PLoS Genet. 2012 Jul 12;8(7):e1002791. doi: 10.1371/journal.pgen.1002791 (PMC3395595; doi:10.1371/journal.pgen.1002791)
Supplement: Table S9 — Information of primers and probes for the 39 fast-track replicated SNPs of the GWAS scan. (DOCX) [file pgen.1002791.s015.docx]

**Table S9** Information of primers and probes for the 39 fast-track replicated SNPs of the GWAS scan.

| Polymorphism | Sequence(5’-3’) | |
| --- | --- | --- |
| rs7518471 | Primer | F: TAAGAGACTGTCCTTGGGAG |
|  |  | R: ACTGCATGTGCGTGTTTCAG |
|  | Probe | AAATCTTGACACTGAAACCC-SEQ |
|  |  | AAATCTTGACACTGAAACCT-SEQ |
|  |  |  |
| rs7512378 | Primer | F:GGCTCACTGCAGCCTTGAC |
|  |  | R:CCCATCTCTACAGAAATAAAAAATTTACC |
|  | Probe | FAM-TCAGCTTTCTGAGTACC-MGB |
|  |  | HEX:TCAGCTTTCCGAGTAC-MGB |
|  |  |  |
| rs7424161 | Primer | F: ACAGGGTTTTCCATTACTTC |
|  |  | R: GCATACAAAGGTACTTACAAG |
|  | Probe | TTTCTAATAGCTCCTATGTCAC-SEQ |
|  |  | TTTCTAATAGCTCCTATGTCAT-SEQ |
|  |  |  |
| rs1317530 | Primer | F: CCTTTTCTTGAGCTGATTTCC |
|  |  | R: CTAAACTTGTGGCCTTTTAC |
|  | Probe | TTCTGGAGCTGATTTCCTCCTTGTC-SEQ |
|  |  | TTCTGGAGCTGATTTCCTCCTTGTT-SEQ |
|  |  |  |
| rs6721330 | Primer | F:CACACCTACAACTCAAGCATGTGA |
|  |  | R:GAGGCATGAGAACAGGAGAGTGA |
|  | Probe | FAM:AGTCCCTTGCCCACA-MGB |
|  |  | HEX:TCCCTTGCACACACA-MGB |
|  |  |  |
| rs10933971 | Primer | F:CCACCCTCAGAAAAAACTATCACA |
|  |  | R:GCATGATAGAGTAAAACCCTGTCTCA |
|  | Probe | FAM:ACCAACCAGTGCTTT-MGB |
|  |  | HEX:ACCAACCAGTGTTTT-MGB |
|  |  |  |
| rs907314 | Primer | F: GCATATACTTCTCATGGTGG |
|  |  | R: GCAGAGGCTTTTCCTATACC |
|  | Probe | GGACTTAGAAACTTAAGAGGCC-SEQ |
|  |  | GGACTTAGAAACTTAAGAGGCT-SEQ |
|  |  |  |
| rs16877319 | Primer | F:TGCATAAACATTGCTGGGATTT |
|  |  | R:CCAGGCTATATTTGTATTCCTCAAAGT |
|  | Probe | FAM:TTCCTACCTATGTGCCAC-MGB |
|  |  | HEX-TCCTACCTACGTGCCAC-MGB |
|  |  |  |
| rs2964057 | Primer | F: TTTCTTTCGACAGAGGACCC |
|  |  | R: GCCTTGAAGTTCCAGTTGTG |
|  | Probe | GAGGACCCCTGTAAACCCTC-SEQ |
|  |  | GAGGACCCCTGTAAACCCTT-SEQ |
|  |  |  |
| rs9272105 | Primer | F:CAATTGTTTTTATAAGTTTCCTCTGCTTC |
|  |  | R:AGTCCTGTATGCTGATATCCAGTCAC |
|  | Probe | FAM-TGATTGTGAGAGACC-MGB |
|  |  | HEX-TGATTGTGAGAGGCC-MGB |
|  |  |  |
| rs7739131 | Primer | F: GCGAGACCCCTATGTAATAC |
|  |  | R: CAAAGCCTGTGTACTTTGAG |
|  | Probe | GAGAACCCTATGTAATACCATGGTCC-SEQ |
|  |  | GAGAACCCTATGTAATACCATGGTCT-SEQ |
|  |  |  |
| rs9341799 | Primer | F: GACAGTATATGTGGAGAATC |
|  |  | R: CTCTGGTCCTTACAGACTGA |
|  | Probe | GTGGAGAATCAAGCACAGC-SEQ |
|  |  | GTGGAGAATCAAGCACAGT-SEQ |
|  |  |  |
| rs2189638 | Primer | F: TAATCTTGTAAAGTTTTTCC |
|  |  | R: ACAAACAAAAAATCACATAG |
|  | Probe | AATCTTTAATAAGTTTTTAGCATTTTA-SEQ |
|  |  | AATCTTTAATAAGTTTTTAGCATTTTG-SEQ |
|  |  |  |
| rs1465387 | Primer | F:GGCGCTTGTGATTGGGTAAT |
|  |  | R:TGATCTGAGAAAGGAAAGTGAAAGG |
|  | Probe | FAM:ACTCCCTTCTAGCCCT-MGB |
|  |  | HEX:CTCCCTCCTAGCCCT-MGB |
|  |  |  |
| rs1822387 | Primer | F: GGAATAGTACAAGAATTCTCG |
|  |  | R: CTCTCCACTTGCTGTATTCC |
|  | Probe | TGAGAATTCTCGTTTGTTGCA-SEQ |
|  |  | TGAGAATTCTCGTTTGTTGCG-SEQ |
|  |  |  |
| rs7036455 | Primer | F: ACACTCCTTGAGTTCCAACC |
|  |  | R: AAGCCCAGAGATGAGAGAAC |
|  | Probe | GGGGACCCTGCAGTAGCC-SEQ |
|  |  | GGGGACCCTGCAGTAGCT-SEQ |
|  |  |  |
| rs2666261 | Primer | F: ATGTTGAGTCACATCCCTGC |
|  |  | R: GTCTGGAGGCATTTTTGGTC |
|  | Probe | GGGGCGTGGATTCCAGTAGCATCC-SEQ |
|  |  | GGGGCGTGGATTCCAGTAGCATCT-SEQ |
|  |  |  |
| rs12782065 | Primer | F: TCCAACAGCAATAGATGAGG |
|  |  | R: TCAAACAGACAGACAATGAC |
|  | Probe | GTTCTAATTTCTCCATATCCTTGC-SEQ |
|  |  | GTTCTAATTTCTCCATATCCTTGT-SEQ |
|  |  |  |
| rs3825023 | Primer | F:CCCTGTGAGTTCATGTCTAAAGCTT |
|  |  | R:TCTGTGCTGAGAAATATCAGGACATC |
|  | Probe | FAM-CTGCCACATCTCAT-MGB |
|  |  | HEX-CTGCCACATCTTAT-MGB |
|  |  |  |
| rs10160758 | Primer | F: TGAACTGGTCCATATTAGGG |
|  |  | R: CCTGGCAGTCAGACCTTATT |
|  | Probe | GGATGTAATACATGCTACAAAC-SEQ |
|  |  | GGATGTAATACATGCTACAAAT-SEQ |
|  |  |  |
| rs10896464 | Primer | F: ATCCAACTTTGTCTCGGTCC |
|  |  | R: ACAAAGGATGGCTCAGCAAG |
|  | Probe | CTGCAGAGTGACCCAC-SEQ |
|  |  | CTGCAGAGTGACCCAT-SEQ |
|  |  |  |
| rs12364540 | Primer | F: CCTTCGGATGGGAACTATAC |
|  |  | R: GAGGCTGACAAGTCTCAAGA |
|  | Probe | CTATACCGTCAGCTCTTCC-SEQ |
|  |  | CTATACCGTCAGCTCTTCT-SEQ |
|  |  |  |
| rs7977334 | Primer | F: ATTTTCCTCCTCGTCCTAGC |
|  |  | R: GACAACCAAAAACGTATGCAG |
|  | Probe | ATAGCCACTACCTCCTCTA-SEQ |
|  |  | ATAGCCACTACCTCCTCTG-SEQ |
|  |  |  |
| rs11053534 | Primer | F: GCCTGATATGTTTGAAGAAG |
|  |  | R: GCCAGGTCCTGATGTTTTAC |
|  | Probe | GGAGAAGAGAAAGGAATCCAA-SEQ |
|  |  | GGAGAAGAGAAAGGAATCCAG-SEQ |
|  |  |  |
| rs11168830 | Primer | F: AGAGGCTAAAATTGGCGGTG |
|  |  | R: CTTTGTCATCTTGCCCCTTG |
|  | Probe | TGTAGCAGGCTCCGTTAGA-SEQ |
|  |  | TGTAGCAGGCTCCGTTAGG-SEQ |
|  |  |  |
| rs6538797 | Primer | F: ATGTCAAGACTGGACCACAC |
|  |  | R: ACGAGGTCTAAAGGGCATGG |
|  | Probe | GGGGACACACTGTGGCATGGCA-SEQ |
|  |  | GGGGACACACTGTGGCATGGCG-SEQ |
|  |  |  |
| rs7313883 | Primer | F:GCTGCTTTGTTTCCAACATCTCT |
|  |  | R:GCATGCAAATACAGGCAAGACT |
|  | Probe | FAM:ATTGGCACGATGTC-MGB |
|  |  | HEX:CATTGGCATGATGTC-MGB |
|  |  |  |
| rs3129595 | Primer | F: ACTCCAGACTGTCTGGCAC |
|  |  | R: AGAAAGGCGGGATTCTGCTG |
|  | Probe | CTGTCTGGCACATGAACC-SEQ |
|  |  | CTGTCTGGCACATGAACT-SEQ |
|  |  |  |
| rs11148740 | Primer | F:GGGTCAGATGAAAAAGGTTGAAA |
|  |  | R:AGAGGATGAAAGAGCAAAATGAAAA |
|  | Probe | FAM:CTCTAAAGCGCTAAAC-MGB |
|  |  | HEX:CTCTAAAGTGCTAAACC-MGB |
|  |  |  |
| rs7149261 | Primer | F: AAAGTGCCAGAATGCTTGAG |
|  |  | R: GAAGTGAGGTGACTTGCACA |
|  | Probe | GCCTTACTCTATCACTCACTC-SEQ |
|  |  | GCCTTACTCTATCACTCACTA-SEQ |
|  |  |  |
| rs234601 | Primer | F: GATTGTCACCAAACCATCCC |
|  |  | R: AAGCTGAGCAGAAACGCTGG |
|  | Probe | GACCTCCCATAATGAGAAACATCAA-SEQ |
|  |  | GACCTCCCATAATGAGAAACATCAG-SEQ |
|  |  |  |
| rs7197658 | Primer | F: GACAGCGATCTCATCAAGTG |
|  |  | R: GTGTGGGATAAATGAGTTGG |
|  | Probe | AGTAGGCCACTAGGTTG-SEQ |
|  |  | AGTAGGCCACTAGGTTT-SEQ |
|  |  |  |
| rs4151117 | Primer | F: AGGCCAGCCTTGACCATTCT |
|  |  | R: AGAGGAGAGTTTTGGGGCTG |
|  | Probe | TGACCATTCTCCACCTG-SEQ |
|  |  | TGACCATTCTCCACCTT-SEQ |
|  |  |  |
| rs7215223 | Primer | F: CAGTTTACACAGTCCTCTGC |
|  |  | R: TGGACTCATAGGAGTTCCTG |
|  | Probe | GTGTGGTTAATCCCCTC-SEQ |
|  |  | GTGTGGTTAATCCCCTT-SEQ |
|  |  |  |
| rs17305346 | Primer | F:GAGAAGACACGTTCTAATTCCACTGT |
|  |  | R:CCACATGAATGCAACCACACA |
|  | Probe | FAM:CTGAGTACTCACATGTGTA-MGB |
|  |  | HEX:CTGAGTACTCATATGTGTAC-MGB |
|  |  |  |
| rs3092194 | Primer | F: ATAAAGGAGGTAACGCTCAT |
|  |  | R: CTATGGGGCCAGGATTTAG |
|  | Probe | CCGCTCATTTTACCAGGAACA-SEQ |
|  |  | CCGCTCATTTTACCAGGAACG-SEQ |
|  |  |  |
| rs7262054 | Primer | F: CTGGAAGGGAGGTGCAATAA |
|  |  | R: AAAGTGACTTCTCCCCTGTG |
|  | Probe | ATGTGCAATAATTATCCTTGTCTC-SEQ |
|  |  | ATGTGCAATAATTATCCTTGTCTT-SEQ |
|  |  |  |
| rs455804 | Primer | F:TCAACATCTCAGCACATTTGTAACAT |
|  |  | R:GAAGGTGTGTTGTTTTGCTATTATTTTAAG |
|  | Probe | FAM:ACTAAGGCACTTTAAACT-MGB |
|  |  | HEX:CTAAGGCAATTTAAAC-MGB |
|  |  |  |
| rs743417 | Primer | F: ATGTTACCATGACAGGAGAC |
|  |  | R: TTCTGGGAATACCCAAAGGC |
|  | Probe | AAGGAGACAAACACATGGCAA-SEQ |
|  |  | AAGGAGACAAACACATGGCAG-SEQ |
